# Targeting Ubiquitin-Proteasome Pathway by Natural Products: Novel Therapeutic Strategy for Treatment of Neurodegenerative Diseases

**DOI:** 10.3389/fphys.2020.00361

**Published:** 2020-04-28

**Authors:** Saeideh Momtaz, Zahra Memariani, Fardous F. El-Senduny, Nima Sanadgol, Fereshteh Golab, Majid Katebi, Amir Hossein Abdolghaffari, Mohammad Hosein Farzaei, Mohammad Abdollahi

**Affiliations:** ^1^Medicinal Plants Research Center, Institute of Medicinal Plants, ACECR, Karaj, Iran; ^2^Pharmaceutical Sciences Research Center (PSRC), The Institute of Pharmaceutical Sciences (TIPS), Tehran University of Medical Sciences, Tehran, Iran; ^3^Gastrointestinal Pharmacology Interest Group, Universal Scientific Education and Research Network, Tehran, Iran; ^4^Traditional Medicine and History of Medical Sciences Research Center, Health Research Center, Babol University of Medical Sciences, Babol, Iran; ^5^Chemistry Department, Faculty of Science, Mansoura University, Mansoura, Egypt; ^6^Department of Biology, Faculty of Sciences, University of Zabol, Zabol, Iran; ^7^Department of Biomolecular Sciences, School of Pharmaceutical Sciences, University of São Paulo, Ribeirão Preto, Brazil; ^8^Cellular and Molecular Research Center, Iran University of Medical Science, Tehran, Iran; ^9^Department of Anatomy, Faculty of Medicine, Hormozgan University of Medical Sciences, Hormozgan, Iran; ^10^Department of Toxicology & Pharmacology, Faculty of Pharmacy, Tehran Medical Sciences, Islamic Azad University, Tehran, Iran; ^11^Pharmaceutical Sciences Research Center, Health Institute, Kermanshah University of Medical Sciences, Kermanshah, Iran; ^12^Medical Biology Research Center, Kermanshah University of Medical Sciences, Kermanshah, Iran; ^13^Department of Toxicology and Pharmacology, Faculty of Pharmacy, Tehran University of Medical Sciences, Tehran, Iran

**Keywords:** ubiquitin-proteasome pathway, neurodegenerative diseases, Alzheimer's disease, Parkinson's disease, Huntington disease, Amyotrophic lateral sclerosis, Multiple myeloma, polyphenols

## Abstract

Misfolded proteins are the main common feature of neurodegenerative diseases, thereby, normal proteostasis is an important mechanism to regulate the neural survival and the central nervous system functionality. The ubiquitin-proteasome system (UPS) is a non-lysosomal proteolytic pathway involved in numerous normal functions of the nervous system, modulation of neurotransmitter release, synaptic plasticity, and recycling of membrane receptors or degradation of damaged and regulatory intracellular proteins. Aberrant accumulation of intracellular ubiquitin-positive inclusions has been implicated to a variety of neurodegenerative disorders such as Alzheimer's disease (AD), Parkinson's disease (PD), Huntington disease (HD), Amyotrophic Lateral Sclerosis (ALS), and Multiple Myeloma (MM). Genetic mutation in deubiquitinating enzyme could disrupt UPS and results in destructive effects on neuron survival. To date, various agents were characterized with proteasome-inhibitory potential. Proteins of the ubiquitin-proteasome system, and in particular, E3 ubiquitin ligases, may be promising molecular targets for neurodegenerative drug discovery. Phytochemicals, specifically polyphenols (PPs), were reported to act as proteasome-inhibitors or may modulate the proteasome activity. PPs modify the UPS by means of accumulation of ubiquitinated proteins, suppression of neuronal apoptosis, reduction of neurotoxicity, and improvement of synaptic plasticity and transmission. This is the first comprehensive review on the effect of PPs on UPS. Here, we review the recent findings describing various aspects of UPS dysregulation in neurodegenerative disorders. This review attempts to summarize the latest reports on the neuroprotective properties involved in the proper functioning of natural polyphenolic compounds with implication for targeting ubiquitin-proteasome pathway in the neurodegenerative diseases. We highlight the evidence suggesting that polyphenolic compounds have a dose and disorder dependent effects in improving neurological dysfunctions, and so their mechanism of action could stimulate the UPS, induce the protein degradation or inhibit UPS and reduce protein degradation. Future studies should focus on molecular mechanisms by which PPs can interfere this complex regulatory system at specific stages of the disease development and progression.

**Graphical Abstract:**
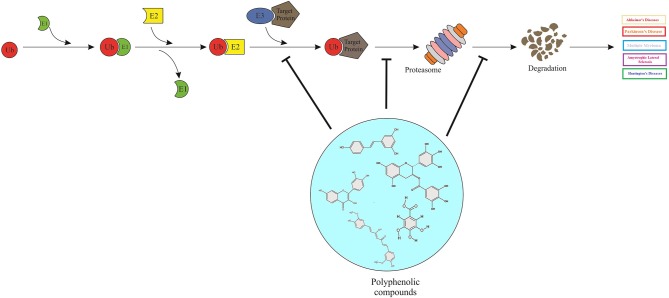
Polyphenols can act as proteasome-inhibitors or may modulate the proteasome activity, thereby improving neurodegenerative disorders by means of accumulation of ubiquitinated proteins, suppression of neuronal apoptosis, reduction of neurotoxicity, and improvement of synaptic plasticity and transmission.

## Highlights

- Aberrant accumulation of intracellular ubiquitin-positive inclusions associated to neurodegenerative disorders.
- Polyphenols, can act as proteasome-inhibitors or may modulate the proteasome activity.
- Polyphenols can manage neurodegenerative impairments by targeting the ubiquitin-proteasome system (UPS).
- Polyphenols exert UPS inhibitory activity, resulting in the accumulation of ubiquitinated proteins, suppression of neuronal apoptosis, reduction of neurotoxicity, and improvement of synaptic plasticity and transmission.

## Introduction

Proper production and degradation of proteins are vital for both cellular homeostasis and neuronal function. Approximately, the majority of cellular proteins are degraded by UPS, highlighting its regulatory effect on cell cycle, proliferation, and survival procedures. Generally, UPS modulates the procedure of proteolysis in neurons. In this manner, an evolutionarily conserved small protein named ubiquitin attached to the substrates (misfolded proteins); under precisely controlled conditions, via the sequential participation of several ubiquitinating and deubiquitinating enzymes, tagging them for degradation by a multi-subunit complex called the proteasome (Hegde and van Leeuwen, 2017). Later, the 26S proteasome binds to the polyubiquitinated proteins and efficiently degrades them (Grice and Nathan, 2016). In addition, proteolysis by UPS has great importance in regulation of many physiological processes, from gene transcription to posttranslational modification of proteins (Hegde and van Leeuwen, 2017).

Emerging data revealed that UPS plays an undeniable role in several neurodegenerative diseases, although, UPS impairment has been reported during the process of neurodegeneration. The proper function of proteasome and UPS are essential factors for specific function of neurons (Jara et al., 2013). The deposition of protein aggregates and the formation of inclusion bodies are common features of most neurodegenerative diseases, since, the majority of intracellular inclusions contain ubiquitin. For instance, mutant α-synuclein (SACN) protein in PD, Amyloid-β (Aβ) and tau protein in AD, expanded polyglutamine tracts in HD and misfolded prion protein in Prion disorders (Zheng et al., 2016); besides, UPS dysregulation results in increased endoplasmic reticulum stress, and cell death in neuronal cells (Jara et al., 2013). In addition, current pharmacological standard treatments for neurodegenerative diseases, such as cholinesterase inhibitors licensed to treat AD; dopaminergic treatments for PD; antidepressants, neuroleptics, and tetrabenazine for HD; glutamate blockers to treat ALS; elicit a wide range of side effects. In this context, identification and characterization of compounds that selectively inhibit UPS and/or proteasome functions, or the substrates capable of triggering deubiquitinases, gained much attention, particularly in line of drug discovery for neurodegenerative diseases and various cancers.

Accordingly, herbal based interventions, predominantly PPs, seems to be an alternative adjuvant therapeutic approach to delay the onset of neurodegeneration and to reduce the burden of symptoms, to maximize the function and optimize the quality of patient's life. Mechanistic studies on the neuroprotective/neuroregenerative effects of PPs, exhibited that these compounds persuasively act as anti-inflammatory agents and antioxidants, either by quenching free radical species or by inhibiting pro-oxidant enzymes. PPs also function as modulators of the anti-apoptotic factors expression; intracellular neuronal signaling and metabolism; cell survival/death genes; protein aggregation and degradation pathways; and have mitochondrial function either directly or by regulating the mitochondrial signaling pathways (Mandel et al., 2011; Branquinho Andrade et al., 2016; Nabavi et al., 2018b).

Recently, both scientific and public interests shifted toward dietary regimens and nutraceuticals associated with reduced risk of neurodegenerative diseases, in a way to find molecules exploitable for prevention of the onset, progression, and severity of such impairments. Reliable evidence supports the beneficial effects of natural phytochemicals, in particular polyphenolic compounds in attenuating neurological deterioration by means of protein clearance machineries. Among these systems, UPS plays a crucial role in degradation of misfolded protein aggregates. Thereby, the scope of this review is to introduce the UPS in neurodegenerative diseases, to assess the favorable effects of PPs in UPS inhibition, and to discuss their potential application in clinical trials to target neurodegeneration pathologies in the quest for a disease modifying therapy.

## Ubiquitin-proteasome System

The ubiquitin proteasome system (UPS), a 76-amino acid complex, is a key regulator of protein catabolism in the mammalian nucleus and cytosol. The UPS is essential for the regulation of almost all vital processes including, organelles biogenesis, cell cycle, differentiation and development, immune response and inflammation, neural and muscular degeneration, as well as response to stress and extracellular modulators. Under extremely controlled conditions, UPS affects a wide variety of cellular substrates and molecular pathways; furthermore, UPS defects could result in the pathogenesis of numerous devastating human diseases (Leestemaker and Ovaa, 2017).

Protein degradation via UPS involves two separate and consecutive phases named conjugation and degradation. Throughout the conjugation phase, the substrate protein is tagged by the covalent attachment of multiple ubiquitin molecules, thereafter, 26S proteasome (composed of the catalytic 20S core and the regulatory 19S part) degrades the tagged protein, such process is called the degradation phase. This conventional function of UPS is usually associated with antigenic peptide generation, regulation of protein turnover, and housekeeping functions. Recently, it has been shown that protein modification by UPS also has unconventional (non-degradative) functions that is dictated by the number of ubiquitin units covalently tagged to proteins (poly vs. mono-ubiquitination), and by the type of ubiquitin chain linkage that is present (Akutsu et al., 2016). Ubiquitin is tagged to the ε-amine of lysine residues of target proteins via a series of ATP-dependent enzymatic steps named; E1 (ubiquitin activating), E2 (ubiquitin conjugating), and E3 (ubiquitin ligating) enzymes. Moreover, the C-terminal Gly75-Gly76 residues of ubiquitin are the key residues that play critical roles in the diverse chemistry of ubiquitin reactions. Ubiquitin could be conjugated to itself through particular lysine residues (K6, K11, K27, K29, K33, K48, or K63), resulting in various types of chain linkages. The isopeptide binds to a target protein and ubiquitin, thereby; specific deubiquitinating enzymes (DUBs) can reverse the linkages between several ubiquitins in a chain. Recent studies have revealed that many DUBs are parts of ubiquitin ligase complexes, which enables DUBs to regulate the activity and abundance of both the ligase and the substrate (Stolz and Dikic, 2018).

Substantial evidence has clarified the determinative role of UPS dysfunction in neurodegenerative disorders, involving abnormal accumulation of inclusion bodies or insoluble protein aggregates in neurons. Furthermore, dysregulation of UPS could impede the degradation of aberrant or misfolded proteins and negatively upset synaptic transmission (Zheng et al., 2014). Eventually, unsuccessful removal of damaged proteins could result in the aggregation of these proteins and neuronal apoptosis (Hyttinen et al., 2014). On the other hand, in neurodegenerative disorders, defectiveness in synaptic plasticity is attributed to dysregulation of ubiquitin-mediated proteolysis (Selkoe, 2002).

## Deubiquitation

DUBs play pivotal roles in hemostasis of biological processes such as cell cycle, proliferation, programmed cell death apoptosis, and DNA repair mechanisms. Ubiquitin-specific-proteasome-7 is a DUB enzyme. Its overexpression has been detected in numerous types of cancers (Hu et al., 2002; Li et al., 2004; Nicholson et al., 2007), in particular, stabilization of MDM-2 (murine double minute). USP7 deubiquinates MDM2, therefore, maintaining the tumor suppressor p53 ubiquitinated, it also degraded by proteasome under normal condition (Everett et al., 1997; Cummins et al., 2004). In MM patients, deletion or mutation in p53 was detected, thereby; the inhibition of USP7 could be a useful therapeutic target for accumulation of functional p53.

Both USP14 ubiquitin-specific-protease and the ubiquitin C-terminal hydrolyase (UCHL5) are cysteine proteases. DUBs are associated with 19S proteasome regulatory subunit; hence, they may modulate the capability of proteasome for target proteins to be degraded (Borodovsky et al., 2001; Al-Shami et al., 2010; Lee et al., 2010). They are able to regulate the signaling pathways such as nuclear factor (NF)-κB (Al-Shami et al., 2010), transforming growth factor (TGF)-β (Wicks et al., 2005), and CXCR4 chemotaxis (Mines et al., 2009). USP14 and UCHL5 expression levels are upregulated in different types of tumors such as colorectal cancer (Shinji et al., 2006), ovarian cancer and MM (Tian et al., 2013). Selective USP14 and UCHL5 inhibitor b-AP15, induced apoptosis in MM cell lines and in primary MM cells via downregulation of cell division cycle 25C (CDC25C), CDC2, and cyclin-B1, as well as the activation of caspases and unfolded protein response pathways (p-IREα, p-eIF2α, and CHOP) (Tian et al., 2013). VLX1570 is another USP14 inhibitor, which induced apoptosis in MM cells (Wang et al., 2016).

## Alzheimer's Disease

Alzheimer's disease (AD) is recognized as a highly common neurodegenerative disease with visual-spatial confusion and loss of short-term memory. It is known that memory loss exacerbates over time, leading to cognitive dysfunction and reduced intellectual capacity in AD patients. The pathology of AD is related to misfolded-protein aggregation, inflammatory changes, and oxidative damage, resulting in neuronal loss (Querfurth and LaFerla, 2010). In an AD brain, the most important pathognomonic lesions include the intracellular neurofibrillary tangles (NFTs) and extracellular senile plaques (ESPs). Senile or neuritic plaques [composed of Aβ containing 39 to 42 amino-acid peptides, a product of the sequential cleavage of the β-amyloid precursor protein (APP)] and neurofibrillary tangles (filamentous bundles comprised of hyperphosphorylated tau proteins) are typical characteristic lesions in affected tissues (Haass and Selkoe, 2007). To date, two types of medications including cholinesterase inhibitors (Donepezil, Rivastigmine, Galantamine), and *N*-methyl-D-aspartate (NMDA) receptor antagonist (memantine) were approved by the U.S. Food and Drug Administration (FDA) to treat moderate to severe AD symptoms (Briggs et al., 2016).

Although the major cause of AD remains unknown, the familial type of autosomal dominant inheritance is involved in nearly 0.1% of cases. Mutations in genes encoding presenilin 1 (PS1), PS2, and amyloid precursor protein (APP), are incorporated to this type of AD (Waring and Rosenberg, 2008). Nearly 95% of all AD patients suffer from sporadic AD, which is associated with the late onset of symptoms (above 65 years) (Minati et al., 2009). According to a recent investigation, UPS either is damaged or appears inadequate in some regions of the brain of young mice (Liu et al., 2014). In this regard, the interaction of ubiquitin C with different AD factors was reported by a proteomic study; accordingly, UPS dysregulation was introduced as a mechanism underlying AD (Manavalan et al., 2013). UPS dysregulation can prevent calmodulin degradation and block Ca^2+^/calmodulin-dependent signaling pathways in AD (Esteras et al., 2012). Some UPS and AD-related proteins, such as C-terminus of Hsc70-interacting protein (CHIP) and ubiquitin carboxyl terminal esterase L1 (UCHL1), may be expressed aberrantly. These proteins along with a mutant form of ubiquitin, can inhibit the UPS and cause proteasomal dysfunction in AD patients (Oddo, 2008; Bilguvar et al., 2013).

### Ubiquitin-Proteasome System and Amyloid Beta

Aβ is identified as a peptide from APP, cleaved by β- and γ-secretases. Following cleavage of APP in its ectodomain by β -secretase 1 (BACE1), γ-secretase splits the transmembrane domain of carboxy-terminal fragments and discharges Aβ peptides into the extracellular environment (Wang et al., 2018). Overexpression of APP increased the activity of UPS in the frontal cortex of transgenic AD mice model (Seo and Isacson, 2010). According to the literature, Lysine (Lys)-203 and Lys-382 are indispensable to proteasomal degradation of BACE1 (Wang et al., 2012). On the other hand, BACE1 proteasomal degradation is accelerated by ubiquitin carboxylterminal hydrolase L1 (UCHL1) (Zhang et al., 2012). It was shown that due to the accumulation of Aβ in neurons, the activities of proteasomes and the deubiquitinating enzymes reduced (Almeida et al., 2006). Clearance of Aβ can significantly diminish the early pathogenesis of tau (Budd Haeberlein et al., 2017). However, Aβ accumulation may damage proteasome function and promote tau accumulation (Tseng et al., 2008). In addition, mutant or wild-type APP in neural cells is known to affect downstream protease inhibition (Cecarini et al., 2014).

### Ubiquitin-Proteasome System and Tau

Tau, which is described as a soluble protein in neurons, is concentrated in axons and stabilizes the microtubule network (Lee et al., 2013). In adult human brain, six tau isoforms are expressed. Although, the mechanism of tau fibrillization is still indefinite in pathological disorders, formation of paired helical filaments (PHFs) is triggered by hexapeptide motifs. Overall, diverse posttranslational modifications such as glycosylation, ubiquitination, hyperphosphorylation, and proteolysis could occur in tau (Hernandez and Avila, 2007; Martin et al., 2011). Besides, tau hexapeptide motifs, ubiquitin and apolipoprotein E are among other NFT components. Stepwise fragmentation happens in tau to generate cleaved molecules with proaggregation features, such as neurodegeneration (Wang et al., 2010). In a study by Dolan and Johnson, the autophagy system removed truncated tau, while UPS was not involved (Dolan and Johnson, 2010). On the other hand, Grune et al. (2010) reported that ATP/ubiquitin-independent 20S proteasome catalyzed tau degradation. Valosin-containing proteins (part of UPS; the machinery that degrades damaged, misshapen, and excess proteins within cells) in AD synapses are negatively correlated with the buildup of hyperphosphorylated tau oligomers, and UPS dysfunction may progress concomitantly with tau hyperphosphorylation (Tai et al., 2012).

### Ubiquitin-Proteasome System and Ubiquitin Carboxyl Terminal Esterase L1

UCHL1 enzyme attributed to the removal of ubiquitin from unfolded proteins and disassembly of polyubiquitin chains for recycling of ubiquitin. The enzyme is also capable of eliminating abnormal proteins, as it stabilizes monoubiquitinated proteins (Setsuie and Wada, 2007). In a model of APP/PS1 mice, UCHL1 transduction restored normal cognition and synaptic function in hippocampal slices treated with Aβ (Gong et al., 2006). The direct correlation of neuronal UPS with sporadic AD has been proven (Oddo et al., 2006). In a study on Chinese Han patients, AD was associated with serine-to-tyrosine substitution at codon 18 in exon 3 of UCHL1 gene; the genotypes were also more resistant in females (Xue and Jia, 2006). According to a recent study by Poon et al., UCHL1 was recognized vital for the regulation of neurotrophin receptors and supporting retrograde transport. It is also known that Aβ downregulates the UCHL1 in AD, thereby, compromising synaptic plasticity, as well as neuronal survival (Poon et al., 2013).

### Ubiquitin-Proteasome System and Ubiquilin-1

Polyubiquitinated proteins are delivered to proteasomes for degradation by several proteins, including ubiquilin-1 with ubiquitin-like domains. The increased risk of AD is associated with the ubiquilin-1 gene (*UBQLN1*) allelic variant (Li et al., 2017). In a study by Stieren et al., reduction of ubiquilin-1 activity, led to the production of APP fragments, along with neuronal cell death (Stieren et al., 2011). Ubiquilin-1 seems to also contribute to the pathogenesis of other neurodegenerative disorders (Safren et al., 2015).

### Ubiquitin-Proteasome System and Sequestosome 1 (p62)

Most NFTs contain p62, which is a UPS-related protein (Morawe et al., 2012). P62 serves as a receptor to bind ubiquitinated proteins and to shuttle proteins to proteasome for the purpose of degradation (Zaffagnini et al., 2018). Similarly, p62 shuttles polyubiquitinated tau to proteasome. In AD, p62 is detected in neuronal inclusion bodies, containing aggregates of ubiquitinated protein (Salminen et al., 2012).

## Parkinson Diseases

PD is associated with severe motor symptoms, attributing to dopaminergic neuron death in the substantia nigra (Kaur et al., 2018). A number of medications have been approved to treat PD symptoms, of which Levodopa is the most effective pharmacologic treatment for severe motor symptoms, moreover, monoamine oxidase type B (MAO-B) inhibitors, dopamine agonists (i.e., Bromocriptine, Pergolide, Pramipexole, Ropinirole) are effective for patients with mild symptoms at a younger age (Jankovic and Poewe, 2012). Of course, patients using such drugs are facing a verity of complicated adverse effects. Aside from reduced function of UPS, oxidative stress, and mitochondrial metabolism impairment seem to be involved in PD pathogenesis (Winklhofer and Haass, 2010). PD is associated with 10 different mutations, some of which is correlated with UPS, such as alpha-synuclein (α-SNCA), protein deglycase DJ-1 (or PARK7), UCHL1, PTEN-induced kinase 1 (PINK1), and PD protein 2 (PARK2 or parkin). Overall, parkin, PINK1, and DJ-1 mutations are involved in the autosomal recessive familial type of PD (Lunati et al., 2018; Zeng et al., 2018).

### Ubiquitin-Proteasome System and α-synuclein

α-SNCA is described as the major constituent of Lewy bodies (LBs) in the brain of PD patients. LBs contain ubiquitinated proteins, such as α-SNCA. The LB protofibrils exert inhibitory effects against the degradation of 26S proteasome-mediated proteins (Chen et al., 2006; Zhang et al., 2008). α-SNCA is encoded by SNCA gene. Several mutations in SNCA at A53T, and A30P are directly linked to the familial form of PD and α-SNCAopathies (Kaur et al., 2018). It was shown that proteasome inhibitors cause α-SNCA aggregation and formation of LBs (Banerjee et al., 2014). Moreover, rats exposed to proteasome inhibitors displayed PD-like behavior and damage to the central nervous system similar to that observed in PD patients (Lorenc-Koci et al., 2011). Meanwhile, the α-SNCA aggregations may in turn selectively bind to the 6S subunit of the 26S proteasome to inhibit the proteasome activity, and to further induce neurons cytotoxicity, including mitochondrial damage and apoptosis (Tanaka et al., 2001; Snyder et al., 2003). Therefore, proteasome inhibition and α-SNCA may reciprocally regulate a feed forward mechanism and exacerbate the development of PD (Xie and Wu, 2016).

### Ubiquitin-Proteasome System and Protein Deglycase DJ-1

Although DJ-1 protein is majorly expressed in the cytosol, it can also be detected in the nucleus. According to a study by Khasnavis et al., astrocytes produce DJ-1 in mice brain (Khasnavis and Pahan, 2014). Similarly, in a normal human brain, the astrocytes express DJ-1 (van Horssen et al., 2010). As suggested in literature, patients with sporadic PD have reduced levels of DJ-1 protein in the substantia nigra (Nural et al., 2009; Cookson and Bandmann, 2010). Familial forms of PD is associated with DJ-1 mutations (Giguere et al., 2018). In a study by Xiong et al., DJ-1 deficiency reduced parkin ubiquitination and improved aggregation of misfolded parkin substrates (Xiong et al., 2009). Nonetheless, to maintain the mitochondrial function, DJ-1 acts along with the PINK1/parkin pathway. Therefore, the association between DJ-1 and PINK1/parkin should be confirmed in further studies. Moreover, DJ-1 is described as a substrate for small ubiquitin-like modifier-1 **(**SUMO-1) conjugation, and its simulation is crucial (Shinbo et al., 2006).

### Ubiquitin-Proteasome System and PTEN-Induced Kinase 1

PINK1 is expressed in different brain regions, including the hippocampus and substantia nigra (Blackinton et al., 2007; Heeman et al., 2011). Degradation of heat-induced misfolded proteins, mediated by parkin, is increased by the PINK1 expression. On the contrary, parkin and PINK1 mutations in PD, are less potent in promoting parkin substrate degradation. In fact, PINK1 leads to the clearance of aberrant proteins via proteasomal degradation (Clements et al., 2006). PINK1 mutations are involved in some cases of autosomal recessive and sporadic PD (Blackinton et al., 2007). The symptoms of PINK1 knockout mice, including mitochondrial dysfunction and reduced corticostriatal synaptic plasticity in dopaminergic neurons, are similar to PD patients (Kitada et al., 2007, 2009). Liu and colleagues reported that PINK1 deficiency interrupts proteasome activity and leads to α-SNCA aggregation. They also suggested a relationship between PINK1 and UPS in PD (Liu et al., 2009).

### Ubiquitin-Proteasome System and Parkin

Considering the direct correlation between UPS and PD, the parkin gene mutations have been suggested to be involved. The amino acid sequence of parkin contains an ubiquitin-like domain at the N-terminus, as well as a RING box at the C-terminus with E3 ubiquitin-ligase activity (Kaur et al., 2018). Respecting T240R mutations in parkin gene, the association between autosomal recessive familial PD and parkin was identified. In addition, parkin can be found in PD-affected brain regions. Parkin exhibits neuroprotective functions in PD, which can be attributed to its E3 ubiquitin-ligase activity (Song et al., 2009). Wild-type α-SNCA is the most important target of ubiquitin degradation (Li et al., 2018). Research on the possible association of parkin with neurodegeneration reveals that parkin can regulate the aggresome-autophagy pathway (Lim et al., 2006). In addition, parkin triggers ubiquitination, as well as polyglutamine-expanded ataxin-3 degradation, resulting in reduced toxicity in cells (Kumar et al., 2012; Zheng et al., 2014). The deubiquitinating enzyme activity of ataxin-3, as a deubiquitinating enzyme, is improved through ubiquitination (Todi et al., 2009). To eliminate misfolded proteins, ataxin-3 and parkin contribute to aggresome formation (Olzmann et al., 2008). Parkin gene mutations lead to abnormal toxic substrate aggregation due to UPS dysfunction. Parkin might also be associated with the pathogenesis of PD, considering its role in mitochondrial functioning (Kumar et al., 2012). In addition, many mitochondrial processes in PD, involving parkin, are interrupted.

## Multiple Myeloma

Multiple myeloma (MM) is a hematologic malignancy of bone marrow characterized by the accumulation and infiltration of mature plasma cells in the bone marrow (cells that produce high level of antibodies) (Morgan et al., 2012). The cancer incidence is low around 1, in every 132 individuals (0.76%). According to American cancer society, around 30,770 new cases will be diagnosed (16,400 in men and 14,370 in women) with MM and around 12,770 deaths will be expected (6,830 in men and 5,940 in women).

The offered treatment regimens for MM patients mainly include chemotherapy with a response range of 40 to 70%. Unfortunately, most patients suffer from relapsing due to the recurrence of the disease. Bone marrow transplantation combined with chemotherapy (Attal et al., 1996) is another regimen; still patients suffer from the recurrence of the cancer (Attal et al., 1996; Mitsiades et al., 2002). The urine and serum of patients contains high level of monocolonal immunoglobulins called M-protein or paraprotein, which is consisted of a heavy (most often IgG or IgA but also IgM, IgD, or IgE) and a light chain of kappa or lambda. In some patients, the plasma cells only produce light chain immunoglobulins, which dose not bind to the heavy chain. The light chain immunoglobulins are normally excreted in the urine, although their levels in the urineare considered as a prognostic marker for the MM patient.

### Proteasome in Multiple Myeloma

Production of high amount of immunoglobulins require functional endoplasmic reticulum (ER) and ER quality control system (Ibba and Söll, 1999; Wickner et al., 1999), which prevents the processing of misfolded proteins and their translocation to its distinct location. Unfolded proteins induce the activation of unfolded protein response (UPR) pathway, leading to inhibition of further protein synthesis, and the expression of chaperones and enzymes required for folding proteins. Once, proteins cannot be refolded, misfolded proteins will be tagged for proteasome degradation by 26S proteasome (Ellgaard et al., 1999), however, unfolded protein stress is intolerable and unfixable, the cells will undergo apoptosis (Zinszner et al., 1998; Brewer and Diehl, 2000). Consequently, inhibition of UPR is a chemotherapeutic target in MM cells.

It was found that MM cells and the primary cells from MM patients express high level of UPR genes (i.e., chaperones glucose-regulated protein 78/binding-immunoglobulin protein (GRP78/Bip) and GRP94/gp96) (Obeng et al., 2006). Proteasome 26S is playing a vital role in maintaining cellular hemostasis via degradation of misfolded proteins, regulation of stress responses, cell cycle, DNA repair pathway, and apoptosis (Ciechanover, 2005). Proteasome 20S is a multi-catalytic enzyme with multi subunits. Each subunit performs one of the classical proteolytic activities; either chymotrypsin-like (ChT-L) activity localized in β5 subunit, trypsin-like (T-L) in β2 subunit, or peptidylglutamyl-peptide hydrolyzing (PGPH) activity in β1 subunit (Orlowski and Wilk, 2000).

There are two isoforms of proteasome, constitutive and immunoproteasome. The constitutive proteasome is distributed in most cells, while the immunoproteasome is expressed in cells of lymphoid organs. Immuoproteasome has great importance in antigen presentation by major histocompatibility class I (Rock et al., 1994, 2002), and also in proteolysis of proteins (Rivett and Hearn, 2004). The level of circulating proteasome was examined in 50 patients with MM, which showed a positive correlation with the advanced stages of the disease (Jakob et al., 2007); representative of chemotherapeutic potential of proteasome in MM. The NFκB signaling pathway plays a major role in hemostasis of B-cells progenitor, where it inhibits the assembly of Igκ gene in prematured B-lymphocytes (Scherer et al., 1996), protects B cell lymphoma from apoptosis (Wu et al., 1996), induces the stimulus-dependent proliferation (William et al., 1995), and B cell receptor (BCR) responses (Bendall et al., 1999).

**Figure 1:**
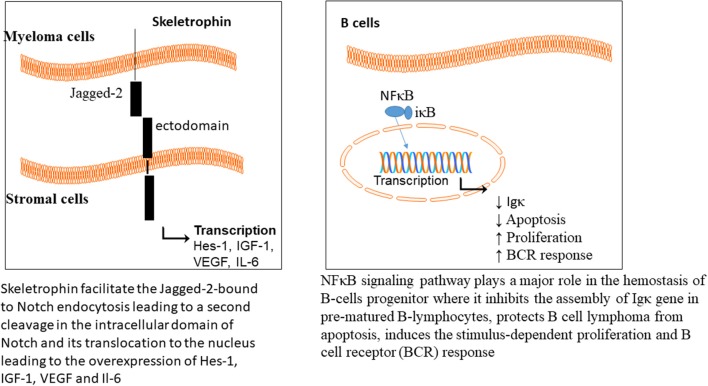
Clinically tested drugs against MM and their targets.

The cancer cells are characterized by high proliferative rate coupled with high levels of misfolded proteins, DNA damage and stress in comparison with normal cells, thus, cancer cells highly require a functional proteasome system. Bortezomib is a proteasomal inhibitor of MM, approved by FDA (Richardson et al., 2003). Bortezomib suppresses the NFκB signaling pathway through inhibition of proteasome degradation of iκB, which can maintain the NFκB sequestered and latent in the cytoplasm (Brockman et al., 1995; Hideshima et al., 2001; Russo et al., 2001; Sunwoo et al., 2001; Ma et al., 2003), activation of p53-mediated apoptosis, cell cycle arrest, and induction of both intrinsic and extrinsic apoptotic pathways. It also activates apoptosis via the c-Jun amino-terminal kinase (JNK)-dependent induction of mitochondrial release of second mitochondria-derived activator of caspase (SMAC) (Chauhan et al., 2003). The resistance of MM cells toward bortezomib, initiates an urgent need for either alternative or combinatorial therapy for MM patients (Figure 1).

### Ubiquitin in Multiple Myeloma

CRL4 (Cullin-RING ubiquitin ligase) is an E3 ubiquitin ligase, composed of three subunits DDB1, cullin 4 (CUL4), and regulator of cullins 1 (RBX1/ROC1). This enzyme has great importance in ubiquitination of different cellular proteins such as c-MYC, interferon regulatory factor 4 (IRF4), Ikaros (IKZF1), and Aiolos (IKZF3) transcription factors. Lenalidomide treatment led to targeted degradation of IKZF1 and IKZF3 in different MM cell lines and primary cells from patients, via inhibition of CLR4 E3 ligase enzyme (Krönke et al., 2013).

**Figure 2:**
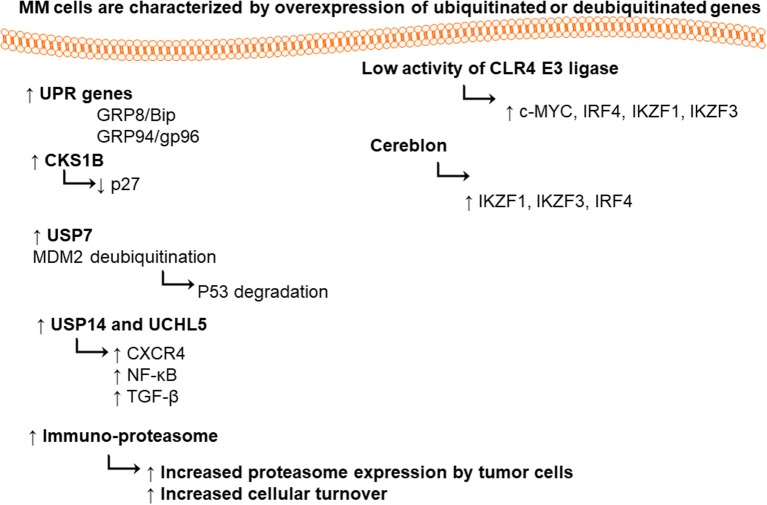
Ubiquitinated or deubiquitinated genes in MM cells.

Cereblon is a substrate recognizing subunit in E3 ligase enzyme, which binds to the DNA damage binding protein-1 (DDB1), CUL4, and Roc1 to form a functional enzyme complex (Fischer et al., 2014; Sang et al., 2015). Cereblon (442 amino acid protein) is ubiquity expressed in plants, rats, mice as well as humans, and is known to be responsible for memory and intelligence in humans (Higgins et al., 2010). Deletion of C-terminal of cereblon due to a non-sense mutation at amino acid 419 (R419X), resulted in intellectual disability syndrome. Cereblon binds to potassium (Higgins et al., 2008) and chloride channels in the brain and retina (Jo et al., 2005; Hohberger and Enz, 2009; Aizawa et al., 2011), respectively. It also inhibited the activation of adenosine monophosphate (AMP) kinase via binding to its subunit α1 (Lee et al., 2011) (Figure 2). Cereblon was found to be a selective target for thalidomide PS-341 (Ito et al., 2010) and lenalidomide (Krönke et al., 2013) activities in MM, leading to the degradation of IKZF3 and IKZF1. The degradation of IKZF3 transcription factor by lenalidomide led to reduction in mRNA and protein levels of IRF4 in multiple myeloma cells. IRF4 is a transcription factor required for the activation of lymphocytes (Mittrücker et al., 1997) and the plasma cells differentiation and maturation (immunoglobulin producing cells) (Klein et al., 2006; Sciammas et al., 2006). Shaffer et al. (2008) showed that IRF4 is not genetically altered in myeloma cells, but it is addicted for the maturation and activation of B cells. Such data highlighted the role of CRL4 and Cereblon in regulating the pathogenesis of MM and could be used as further objects for MM treatment.

**Figure 3:**
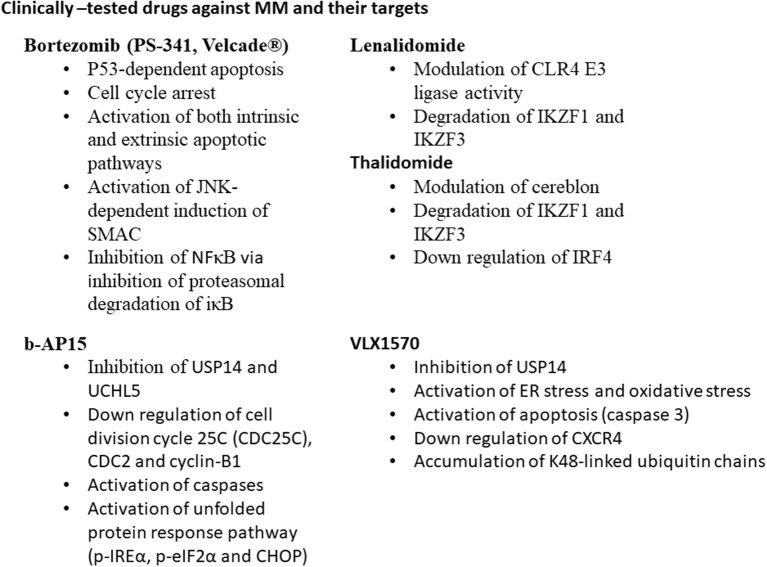
Signaling pathways involved in MM.

Skeletrophin is an E3 ligase, required for Notch signaling pathway activation (Saurin et al., 1996; Freemont, 2000). The Notch extracellular domains are composed of epidermal growth factor (EGF) repeats (29-36), Lin-12/Notch repeat (LIN), linker to the transmembrane and an intracellular domain. The Notch ligands are the transmembrane proteins and divided into two classes, Delta or Delta-like (Dll) and Serrate (Jagged-1 and Jagged-2 in mammals). Upon binding of the ligand to the Notch, a mechanical force leads to the cleavage of the Notch ectodomain, which is followed by endocytosis. Skeletrophin facilitates the Jagged-2-bound to Notch endocytosis, leading to a second cleavage in the intracellular domain of Notch and its translocation to the nucleus (Figure 3).

T-cell acute lymphoblastic leukemia (T-ALL) is characterized by chromosomal translocation, resulting in expression of truncated Notch characterized by constitutive Notch signaling activation. Moreover, myeloma cells as well as primary MM cells express high levels of Jagged-1 and−2, and play a role in the interaction between stromal and myeloma cells (Houde et al., 2004; Jundt et al., 2004). Such data introduce a new ubiquitin ligase, favorable for MM treatment.

CKS1B (Cyclin-Dependent Kinases Regulatory Subunit 1) is an accessory protein in SCF SKP2. F-box (40 amino acids motif)-containing protein S-phase kinase-associated protein 2 (SKP2) is one of the four subunits of ubiquitin ligase complex [SCF (Skp1, Cullin 1, F-box protein)]. SKP2 potentially regulates the transition of cells from G1 to S phase, through a phosphorylation-dependent degradation of cell cycle inhibitor p27 (Cyclin-dependent kinase inhibitor 1B) (Tsvetkov et al., 1999), which is considered as an oncoprotein. The inhibitor p27 downregulation, led to the inhibition of p21 (Yu et al., 1998), p27 (Tsvetkov et al., 1999), and p57 (Kamura et al., 2003) degradation, and prevention of other tumor suppressors such as c-MYC (Kim et al., 2003; Von Der Lehr et al., 2003; Song et al., 2008), transducer of ERBB2, 1 (Tob 1) (Hiramatsu et al., 2006), and Forkhead transcription factors FOXO1 (Huang et al., 2005). The P27 cell cycle inhibitor is also downregulated in numerous cancers including MM (Filipits et al., 2003). In the same time, the CKS1B is overexpressed in oral, gastric, breast and colon cancers, spectating the possible role of CKS1B in regulating the p27 degradation. (Zhan et al., 2007) showed that the silencing of CKS1B led to stabilization of p27 in 4 different MM cell lines.

## Amyotrophic Lateral Sclerosis

ALS is a neurodegenerative disorder, sporadic in most cases, involved in progressive motor neuron degeneration in the brain and spinal cord, associated with muscle weakness (Zheng et al., 2014). ALS characterized by paralysis and death within 3 to 5 years from day of appearance of symptoms due to the impairment of respiratory systems (Hardiman et al., 2011). The loss of bulbar and limb function are the main features of ALS. According to Logroscino et al. (2010) study, the incidence rate in the Europe continent is 2.16/100,000 per year. The incidence rate was higher in men than women. Around 10% of patients inherited the disease (autosomal dominant) and 90% of patients have no family history of ALS (FALS), although they still show the pathologic features of FALS. Until now, there is no effective treatment for ALS, so the survival rate is low for the affected patients (Kim et al., 2009). In this context, only 2 drugs have FDA approval; Riluzole (glutamate inhibitor) and Edaravone (free radical and peroxynitrite scavenger with anti-inflammatory properties), although they might cause side effects such as bruising, gait disturbance, hives, dizziness, gastrointestinal, and liver dysfunctions, as well as allergic reactions (Jaiswal, 2018). Although the cause of ALS is unknown in most cases, several mutations are related to familial ALS, including superoxide dismutase 1 (SOD1), ubiquilin 2 (UBQLN2), and RNA-binding protein fused in sarcoma (FUS) protein (Kwiatkowski et al., 2009; Vance et al., 2009; Deng et al., 2011). Twenty percentage of the familial ALS is linked to a genetic modification in chromosome 21 long arm q (Rosen et al., 1993).

### Amyotrophic Lateral Sclerosis and Superoxide Dismutase 1

Based on several studies, UPS is associated with superoxide dismutase 1 (SOD1) turnover; however, the exact mechanism remains unclear. UPS degrades mutant SOD1 proteins faster than wild-type SOD1, and the proteasome inhibition improves the SOD1 stability (Bendotti et al., 2012). ALS-like pathologies are found in mice with conditional knockout of proteasome subunit, Rpt3 (Tashiro et al., 2012). ALS is also related to the induction of immunoproteasome subunits (Bendotti et al., 2012). In a study using a mutant SOD1-G93A model, pyrrolidine dithiocarbamate treatment reduced the survival of ALS (Ahtoniemi et al., 2007), therefore, the immunoproteasome expression may increase the coping of the nervous system with SOD1 mutations- induced ALS (Rao et al., 2015). In ALS, the function of SOD1 does not decrease, and mice with increased or decreased levels of SOD1 do not show ALS-like pathologies. On the other hand, SOD1 mutation is known to trigger a toxic gain of function, causing SOD1 aggregation. Moreover, in some cases of sporadic ALS, wild-type SOD1 aggregates support the gain of function (Bosco et al., 2010). Mutant SOD1 can transfer the misfolded SOD1, which is followed by degradation (Crippa et al., 2010). In this regard, an increase was reported in the expression level of immunoproteasome (Cheroni et al., 2008). However, according to the literature, autophagy has greater significance in SOD1 turnover, compared to UPS (Dennissen et al., 2012).

In FALS, virtually 11 missense mutations in cytosolic Cu/Zn SOD1 linked to the accumulation of free radicals to the neurodegenerative diseases, where it leads to a damage in the mitochondrial hemostasis, axon transport, and glutamate transporter function (Rosen et al., 1993). It was found that the mutated form of SOD 1 inhibits the chymotrypsin-like activity of proteasome in Neuro2A cells and depresses the induction of motor neuron death in the transgenic mouse model (Urushitani et al., 2002). Kabashi et al. (2004) found that the mutant SOD 1 caused inhibition in chymotrypsin-like, caspase-like and trypsin-like activities of proteasome, without decreasing its level in lumbar spinal cord of the transgenic mice. These data indicated the importance of functional proteasome system in preventing the ALS development.

### Amyotrophic Lateral Sclerosis and Fused in Sarcoma Protein

It was evidenced that genetic modifications such as angiogenin 3 (*ANG*), transactive response (TAR) DNA-binding protein TDP-43 (*TARDP*), sarcoma/translated in liposarcoma and optineurin (OPTN) are linked to FALS. Ubiquitination of misfolded proteins is a pre-step in degradation by proteasome system. The accumulation of misfolded proteins in the hippocampus, neocortex and spinal cord is a pathologic feature of neurodegenerative diseases, TDP-43 protein is composed of glycine-rich C-terminal with two RNA-recognition motifs and it is the main pathologic manifestation for ALS (Wang et al., 2004). Its phosphorylation, ubiquitination and cleavage into two peptide fragments have been linked to the poor prognosis of ALS patients (Leigh et al., 1991; Okamoto et al., 1991; Neumann et al., 2006). It was found that ubiquilin 1 (UBQLN) (proteasome targeting cochaperone factor) binds to the ubiquitinylated TDP-43 aggregates and targets them either to autophagy or proteasomal degradation. The binding of the mutated form of TDP-34 (D169G) to UBQLN was greatly decreased in comparison with the wild-type TDP-43. More studies are required to confirm such hypothesis in the pathogenesis of ALS.

ALS has major similarities to the frontotemporal dementia (FTD) spectrum. FUS and TAR DNA- TDP-43 inclusions are detected in sporadic ALS (Deng et al., 2010). According to the literature, inclusions co-localize with ubiquitin similar to FTD. Moreover, VCP/P97, C9ORF72, and optineurin polymorphisms can produce FALS (Johnson et al., 2010; Maruyama et al., 2010; Deng et al., 2011; Renton et al., 2011). *TARDP* is localized in chromosome 1p36.2. The mutations in *TARDP* gene (p.Gly290Ala and p.Gly298Ser mutations) were found to be linked to the sporadic and FALS (Kabashi et al., 2008). The mutation either leads to gain- or loss-of-function in TDP-43, and may be essential in binding to hnRNPs (heterogeneous nuclear riboproteins) (Van Deerlin et al., 2008). However, the main molecular mechanism of ALS remains undetermined.

### Amyotrophic Lateral Sclerosis and Ubiquilin-2

UBQLN2 disorders involved in the pathogenesis of different neurodegenerative disorders, as this protein regulates ubiquitinated protein degradation. Besides, UBQLN-2 mutations result in FALS, and UBQLN-2 accumulation co-localizes with skein-like inclusions (Deng et al., 2011). UBQLN-2 proteins contribute to the transfer of ubiquitinated proteins to proteasomes. The UBQLN-2 overexpression reduces PS1 and PS2 ubiquitination (Massey et al., 2004). The influence of ALS on lysosomal degradation has been confirmed in a previous study, as UBQLN proteins can increase the binding of early autophagosomes to the lysosomes (N'Diaye et al., 2009).

### Amyotrophic Lateral Sclerosis and Optineurin

Optineurin (*OPTN*) was found to have three different types of mutations in familial and sporadic ALS. The heterozygous Glu478Gly missense mutation ubiquitin-binding region, homozygous Gln398X non-sense mutation and a homozygous deletion of exon 5 (Maruyama et al., 2010). Both missense and non-sense mutations prevented the inhibition of NF-κB (Wagner et al., 2008; Maruyama et al., 2010). Glu478Gly missense mutation induces the accumulation of the mutated protein in the neurons and modulates the formation of inclusion bodies, resulting in a disturbance in the cell biological functions (Maruyama et al., 2010).

### Amyotrophic Lateral Sclerosis and Cyclin F

Williams et al. (2016) performed exome-sequencing for locus chromosome 16p13.3 in order to discover new leads related to ALS pathogenesis. The authors discovered a missense mutation in *CCNF* gene (nucleotide A replaced by G, resulted in amino acid substitution Ser621Gly), which encodes the cyclin F in neuronal cells. Cyclin F (786 amino acid protein) contains F-box motif that recognizes and binds to SKP1 and CUL1 in order to form SCF E3 ligase complex (SKP1-CUL1-F-box protein) (D'Angiolella et al., 2013; Williams et al., 2016). The mutated cyclin F leads to abnormal ubiquitination and aggregation of ubiquitinated proteins such as TDP-43, forming ubiquitinated inclusion (Williams et al., 2016).

### Amyotrophic Lateral Sclerosis and Neural-Precursor-Cell-Expressed-Developmentally-Down-Regulated-8

Ubiquitin-like protein Neural-precursor-cell-expressed-developmentally-down-regulated-8 (NEDD8) has a structure similar to ubiquitins and called Ub-like proteins. Ub-like proteins are classified into two groups according to the manner of protein conjugation; type 1 Ub-like proteins conjugate with the target protein in a way similar but not totally identical to the known ubiquitination mechanism such as NEDD8 and small Ub-related modifier (SUMO1), while type 2 Ub-like proteins have Ub-like protein structure with broad biological functions such Elongin B (subunit of the transcription factor B), Rad23 (Radiation sensitive), and Parkin (Parkinson Protein 2 E3 Ubiquitin Protein Ligase) (Tanaka et al., 1998).

NEDD8 immuno-reactivity was detected in different neurodegenerative diseases such as Parkinson disease (in LBs and Lewy neurites), multiple system atrophy (in ubiquitinated inclusions and oligodendroglial inclusions), AD (in neurofibrillary tangles), motor neuron disease (in ubiquitinated inclusions), and in triplet repeat diseases (in intranuclear inclusions) (Mori et al., 2005). Moreover, its immune-reactivity was also detected in other diseases such as alcoholic liver disease and astrocytoma (in Rosenthal fibers) (Dil Kuazi et al., 2003).

Signaling proteins and phosphoprotein array study in muscles of 36 ALS patients revealed that there are 17 differentially expressed proteins and phosphoprteins in ALS compared to normal muscle cells. In between, heat shock protein 90 (HSP90) (chaperone), and phospho-retinonlastoma (tumor suppressor, p-Rb at Ser780) were overexpressed, while cyclin-dependent kinase 4 (CDK4) and p-p53 at Ser392 were downregulated (Yin et al., 2012). The accumulation of P53 was detected in the nucleus of lumbar spinal cord of ALS patients. Moreover, the study showed that the immuostaining for p53 was also positive for cell cycle regulators (pRb and E2F-1) and apoptotic proteins (Bax, caspase 8 and caspase 3). P53 stabilization is regulated by MDM2 via ubiquitin-proteasome machinery. ALS is characterized by dysfunction in ubiquitin-proteasome system and this may explain the stabilization of p53 and its translocation into the nucleus of the lumbar spinal cord. P53-MDM2 interaction could be a therapeutic target for improving the survival of ALS patients.

## Huntington Diseases

HD is a progressive hereditary neurodegenerative disorder caused by an autosomal dominant defective gene on chromosome 4 that encodes the huntingtin (HTT) protein, containing a repeating sequence of CAG at the N-terminus of HTT, a protein with an abnormally long polyglutamine (polyQ) sequence. In addition, amyloidogenic mutant huntingtin (mHTT) aggregates were implicated in progression of HD (Popovic et al., 2014). Generally, these neuronal aggregates containing N-terminal fragments of polyQ HTT are located in the striatum and in the cortex of HD patients; either in nuclear or in cytoplasmic regions of affected neurons (DiFiglia et al., 1997). Cognitive decline, behavioral abnormalities and involuntary movements accounted as marked hallmarks of HD; probably caused by both neuronal dysfunction and neuronal cell death; leading to a progressive decline in functional capacity, and ultimately death. Several factors have been associated with HD including alterations in calcium level, IGF signaling, vesicle transport, endoplasmic reticulum maintenance, and autophagy (Martin et al., 2015). Yet, there is no definite cure for HD, although several medications are prescribed to treat movement difficulties (Chorea Huntington) such as monoamine depletors (i.e., Tetrabenazine); or those targeting the atypical behavioral aspects of HD such as antidepressants [i.e., serotonin reuptake inhibitor (SSRI)] and antipsychotic drugs (i.e., Olanzapine) (Chen et al., 2012).

Various *in vitro* and *in vivo* studies ratified that dysfunction in ubiquitin metabolism contributes to the pathogenesis of HD, leading to the accumulation and aggregation of insoluble ubiquitin-containing mHTT (Bennett et al., 2007). Typically, HTT is ubiquitinated at amino acids K6, K9, and K15, resulting in its degradation and decreasing the toxicity of mHTT (Kalchman et al., 1996). The accumulation of K48-, K11-, and K63-linked ubiquitin chains in HD mouse models, and the brains from HD patients was shown to be a common feature of HD (Zucchelli et al., 2011). The fact that mHTT aggregates are abnormally enriched for ubiquitin and contain ubiquitin E3 ligases, confirms that the ubiquitination is a key factor in aggregate formation. Global changes in UPS, alike the accumulation of Lys48-, Lys63-, and Lys11-linked polyubiquitin chains associated with HD pathology (Bennett et al., 2007).

### Ubiquitin-Proteasome System and Tumor Necrosis Factor Receptor Associated Factor 6

Tumor necrosis factor receptor associated factor 6 (TRAF6) is an E3 ubiquitin ligase and was found to be overexpressed in postmortem brains of HD patients. *In vitro* cultured cells, TRAF6 promoted the aggregate formation through mediating atypical ubiquitination of Lys6-, Lys27-, and Lys29-linked chains related to HTT. Both mHTT and TRAF6 were localized to insoluble protein fraction.

## Polyphenols in Neurodegenerative Disorders

Various pharmacological agents have been studied and used to find suitable therapeutic interventions in neurodegenerative diseases; however, some defects have always been associated with such treatments, since the pathophysiology of such impairments is yet to be elucidated. A wide range of phytopharmaceuticals are being explored to improve the effects of commonly used drugs in the treatment of neurogenic disorders (Farzaei et al., 2018a), both via prophylactic and disease controlling approaches (Gorji et al., 2018). Natural products can also provide templates for the development of other drug compounds and to design new effective complex molecules (Babitha et al., 2014). Phytochemicals proven to possess potential neuroprotective effects and are able to protect the central nervous system (CNS) against neuronal injury (Kumar, 2006); hence people who consumed higher amounts of natural functional foods were found to show a lower risk for diseases caused by neuronal damage (Lobo et al., 2010).

PPs are the most abundant natural phytochemicals, capable of protecting neuronal cells in different *in vivo* and *in vitro* models through diverse intracellular targets. Various epidemiological and preclinical investigations confirmed the favorable effects of PPs in neurodegenerative diseases, primarily due to their antioxidant properties and their influence on stress response, mainly through nuclear factor erythroid 2-related factor (Nrf2) signaling, triggering the antioxidant defense machinery (Pandareesh et al., 2015; Farzaei et al., 2018b). In addition, polyphenolic compounds may exert neuroprotective effects involving phosphoinositide 3-kinase (PI3K)/protein kinase B (Akt)/glycogen synthase kinase-3β (GSK-3β) (PI3K/Akt/GSK3β) neuronal survival pathway, through the N-methyl-D-aspartate (NMDA) receptors and by downstream signaling in hippocampus and cognitive deficits through tyrosine receptor kinase β (Trkβ) and brain-derived neurotrophic factor (BDNF) in hippocampus (Srivastava et al., 2018). Generally, natural compounds have shown inhibitory or therapeutic effects on neurodegerative disorders via biological effects such as antioxidant, anti-inflammatory, calcium antagonization, anti-apoptosis, and neurofunctional regulation (Choudhary et al., 2013).

Beside their free radical scavenger properties, the mechanisms by which polyphenolic compounds are able to counteract and prevent neurodegenerative diseases include: (1) anti-inflammatory activity through chromatin remodeling (modulation of both DNA methyl transferase and histone deacetylase activities) and alteration in the expression of related transcription factors such as NF-kB (Rahman and Chung, 2010), and dampening of microgliosis, astrogliosis, and glia-derived pro-inflammatory cytokines (Sundaram and Gowtham, 2012; Peña-Altamira et al., 2017; Sarubbo et al., 2017); (2) improvement of mitochondrial function through stimulating the mitochondrial membrane potential and respiratory chain complex IV, enhancing the mitochondrial complex I-IV enzymatic potential, moderating the mitochondrial free radical production, and increasing endogenous antioxidant defense (Fiorani et al., 2010; Davinelli et al., 2013; Cai et al., 2015; de Oliveira et al., 2015) as well as mitochondrial biogenesis through activation of the AMPK/Sirt1/PGC-1α axis (Ayissi et al., 2014; Cao et al., 2014; de Oliveira et al., 2016; Valenti et al., 2016); (3) antioxidant activity through activation of Nrf2/ARE signaling pathway, increasing the expression of nerve growth factor, glial cell line-derived neurotrophic factor, BDNF, TrkA/B, activation of ERK1/2-CREB-BDNF and Akt/Glycogen synthase kinase 3β signaling pathways (Lin et al., 2010; Bagli et al., 2016; Moosavi et al., 2016; Martínez-Huélamo et al., 2017; Sanadgol et al., 2017).

Anthocyanins (Orhan et al., 2015), proanthocyanidins (Strathearn et al., 2014), stilbenes (Braidy et al., 2016), isoflavons (Devi et al., 2017), and curcumin (Hu et al., 2015), are among the most studied dietary phenolic compounds demonstrating protective effects against AD and PD, while there are also studies showing the potential of S-allylcysteine as organosulfur compounds (Farooqui and Farooqui, 2018) and isothiocyanates such as 6-methylsulfinylhexyl isothiocyanate (6-HITC) and sulforaphane (Giacoppo et al., 2015) to be active as neuroprotective dietary phytochemicals.

These herbal constituents seem to create their neuroprotective effects through mechanisms involving the activation of cellular antioxidant responses including activation of the Nrf2-mediated antioxidant response, stimulation of PGC-1α-mediated mitochondrial biogenesis, and alleviating neuroinflammation evoked by the activation of glial cells (de Rus Jacquet et al., 2017a). Several flovonoids like hesperidin, kaempferol, naringin, and epigallocatechin gallate (EGCG) have also been reported to show efficacy against 3-NP-induced neurotoxicity, which is an extensively used animal model for HD (Dey and De, 2015). Onjisaponin B and trehalose enhanced autophagy as one of therapeutic approach against toxic intracytosolic aggregate-prone mHtt protein in HD (Dey and De, 2015).

A number of PPs can modulate neural toxicity or loss by means of their antioxidant properties. For example, it was shown that a synthesized mitochondria-targeted curcumin (MTC), triphenylphosphonium cation-curcumin, reduced free radicals-induced neurotoxicity and mitochondrial impairments *in vivo* and *in vitro* (Hasan et al., 2019a). Similarly, curcumin and MTC showed protective effects against oxidative damage and cerebellar toxicity induced by rotenone *in vivo*, mainly through decrease of lipid peroxidation, and nitric oxide levels, and reduction of glutathione, SOD, and catalase activities, while enhancing the acetylcholine esterase activity (Hasan et al., 2019b).

Beside flavonoids, various non-flavonoid antioxidant phytochemicals like α-mangostin, curcumin, lycopene, and melatonin, have been reported as effective natural compounds in different HD models (Choudhary et al., 2013). Considering the role of oxidative stress and chronic inflammation in the ALS pathophysiology, natural compounds targeting such stressors are supposed to be effective alone or in combination with other natural/chemical substances to find new therapeutic approach for ALS management (Nabavi et al., 2015).

EGCG, quercetin, quercitrin, and curcumin have been found to be effective in ALS models (Koh et al., 2006; Ip et al., 2017). A 12-month, double-blind, randomized, placebo-controlled study on ALS patients, demonstrated that nanocurcumin combined with riluzole improved survival rate during the trial (Ahmadi et al., 2018).

### Polyphenols, Ubiquitin–Proteasome, and Neurodegenerative Diseases

PPs directly or indirectly interfere with the cellular protein degradation systems including the chaperone-mediated autophagy; the ubiquitin–proteasome degradation pathway; and the lysosome-autophagy system, by eliminating the misfolded and damaged proteins. The accumulation of insoluble protein aggregates is a common mark of neurodegenerative diseases, making PPs a great interest for therapeutic strategies (Hajieva, 2017). On one hand, proteasomal inhibition by PPs would be undesirable in neurodegenerative disorders, and in the other hand, proteasome stimulation and reduction of protein degradation by proteasome inhibitors have shown beneficial consequences and were found to be presumably neuroprotective (del Rosario Campos-Esparza and Adriana Torres-Ramos, 2010; de Rus Jacquet et al., 2017b).

PPs and their derivatives have been shown to inhibit UPS (Nabavi et al., 2018a), yet, a number of limitations have impeded their clinical applications. PPs may target various components of this system through mechanisms involving proteasome inhibition, deubiquitinase activity and/or the activities of E1, E2, or E3, thereby, physiologically affecting the essential proteins and/or by effect on the protein substrates, leading to the imbalanced coordinated intracellular protein homeostasis and the consequent off-target effects (del Rosario Campos-Esparza and Adriana Torres-Ramos, 2010; Liu et al., 2015).

Aforementioned, several PPs were reported to act as proteasome-inhibitors, mainly through chymotrypsin-like activity (on both intracellular 26S and purified 20S proteasome) (Nam et al., 2001; Smith et al., 2002; Kazi et al., 2003; Marambaud et al., 2005; Chen et al., 2007a,b; Chang et al., 2015; Ding et al., 2018). Structure activity relationship studies showed that flavonoids with a hydroxylated B ring and/or unsaturated C ring are potent proteasome inhibitors, of which the carbonyl carbon on the C ring could be the site of nucleophilic attack on the proteasome β5 subunit (Chen et al., 2007a). Anthocyanins and anthocyanidins were reported to possess proteasome inhibitory effects, contributing to their neuroprotective activities (Dreiseitel et al., 2008). Several studies demonstrated that the proteasome inhibitory activities of green tea PPs, are responsible for its anticancer and neuroprotective assets (del Rosario Campos-Esparza and Adriana Torres-Ramos, 2010), for instance, EGCG and its analogs were shown to inhibit the chymotrypsin-like activity of the purified 20S proteasome *in vitro* (Nam et al., 2001; Smith et al., 2002). As an exception, curcumin exhibits a binary function against proteasome. Curcumin at low concentrations activates the proteasome (Jana et al., 2004), while at high doses, the compound suppresses the proteasome activity by enhancing the accumulation of ubiquitinated proteins. In neuro 2a and Hela cells, curcumin inhibited the chymotrypsin, trypsin, and post-glutamyl peptidyl-like protease activity of the proteasome, decreased the free ubiquitin levels and increased the protein polyubiquitination (Jana et al., 2004). The neuroprotective effect of curcumin could also be explained by deubiquitination enzymes that specifically regulate the protein polyubiquitination, through cleavage of ubiquitin from ubiquitin-conjugated protein substrates, preventing molecular aggregation (Reyes-Turcu et al., 2009). Curcumin treatment reduced the activities of deubiquitination enzymes in HeLa cells (Si et al., 2007), also curcumin inhibited the ubiquitin isopeptidase activity (Mullally and Fitzpatrick, 2002). In a recent *in vitro* study, myricetin modulated endogenous levels of quality control E3 ubiquitin ligase E6*-*AP and reduced the misfolded proteins inclusions, resulting in the maintenance of proteostasis (Joshi et al., 2019).

A polyphenol-rich extract from elderflower was shown to suppress neurotoxicity elicited by PD-related symptoms in cortical astrocytes via Nrf2 stabilization and inhibition of Nrf2 degradation mediated by the ubiquitin proteasome pathway. Another way, down-regulation of UPS by elderflower polyphenols induces the Nrf2 activation through up-regulation of macroautophagy pathway (also called the lysosome–autophagy protein degradation pathway), leading to the Nrf2 stabilization (de Rus Jacquet et al., 2017b). Nrf2 is a transcription factor involved in regulating the expression of cellular antioxidant enzymes and the genes encoding cytoprotective proteins (Tambe, 2015). Up-regulation of the Nrf2-mediated cellular antioxidant response (i.e., increase in glutathione synthesis and glutathione metabolites levels), results in alleviation of neurodegeneration in PD (de Rus Jacquet et al., 2017b). Quercetin was shown to induce the expression of proteasome subunits by a similar mechanism (Kwak et al., 2003).

**Figure 4:**
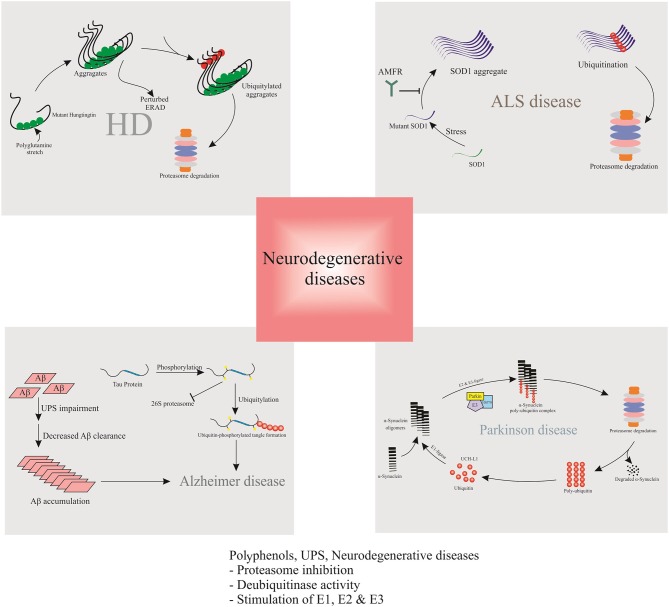
Polyphenols, ubiquitin–proteasome system and neurodegenerative diseases.

In AD brains, Aβ neurotoxicity has been shown to have an inhibitory impact on the activity of UPS (Tseng et al., 2008), compelling a decrease in proteasome activity. In HEK293 and neuro 2a cells transfected with human APP695, resveratrol promoted the intracellular degradation of Aβ in a way that total activity of the proteasome did not increase. This was proved where several proteasome inhibitors such as lactacystin, Z-GPFL-CHO, and YU101 significantly prevented the resveratrol-induced inhibition of Aβ activity, and the siRNA-directed silencing of the proteasome β5 subunit (Marambaud et al., 2005). In mouse model of early PD, an extract of mulberry fruits, rich in phenolic contents (i.e., flavonoids, anthocyanins, and arotenoids), down-regulated the expression of components such as α-SNCA and ubiquitin, also reduced neuronal toxicity; representing that the neuroprotective effect of this plant might be partially mediated by inhibition of the LBs formation in the brain. LBs is thought to trigger dopaminergic neurodegeneration in PD (Gu et al., 2017) (Figure 4).

### Polyphenols/Ubiquitin-Proteasome System Interactions, Clinical Trials

Pharmacological activities of several phytochemicals in neurodegenerative diseases are extensively supported by preclinical and epidemiological studies, which some have shown the mechanistic potential of these compounds as the UP pathway inhibitors (Murakami, 2013). Previously stated, a variety of phenolic compounds have been characterized with their proteasome-inhibitory activity such as resveratrol, EGCG, curcumin, quercetin, chrysin, genistein, kampferol, myrycetin, luteolin, apigenin, gallic acid, ellagic acid, and tannic acid. However, few clinical trials were carried out on a limited basis. Up to date, curcumin and resveratrol are the most studied compounds followed by EGCG and genistein, alone or in combination with other conventional drugs.

#### Curcumin

Currently, 6 clinical trials related to the effects of curcumin on AD is being conducted (http://clinicaltrials.gov/ct2/results?term=alzheimerpmandpmcurcumin), of which 3 have been completed (4, 5, 6) and 2 studies are still in progress, whereas 1 study has unknown status (Table 1). In a pilot randomized clinical trial in China, 34 patients with a diagnosis of probable or possible AD were investigated in 3 subgroups including 4, 1 (compared with 3 g placebo), or 0 g curcumin (compared with 4 g placebo) once daily. They additionally received the standardized extract of gingko leaf (120 mg/day). Compared with the placebo, there was not any significant difference in cognitive function (as secondary outcome) or plasma isoprostanes iPF2α-III and plasma Aβ40 levels (as primary outcome) between 0, 1, and 6 months. Curcumin seemed to cause no major side effects in AD patients in this study (Baum et al., 2008).

**Table 1:** Clinical trials relevant to the phytochemicals with UPS inhibitory activity.

**ID number (www.ClinicalTrials.gov)**	**Phase**	**Medication and dose**	**Duration**	**Number of subjects/Condition**	**Status**	**Country**
NCT00164749	Phase 1,2	Curcumin 1 or 4 g/day (standardized ginkgo extract 120 mg/day)	6 months	34 Probable or possible AD	Published	China
NCT00099710	Phase 2	Curcumin C3 Complex® 2 or 4 g/day (1.9 or 3.8 g/day curcuminoids) ^a^	24 weeks with an open-label extension to 48 weeks	36 Mild-to-moderate AD	Published	USA
NCT00595582	–	Curcumin 5.4 g/day (bioperine)	24 months	10 MCI or mild AD	Completed Results not available	USA
NCT01001637	Phase 2	Longvida® 4 or 6 g/day	2 months	26 Probable AD	Unknown	India
NCT01383161	Phase 2	Theracurmin™ 2.79 g/day (180 mg/day curcumin)	18 months	132 MCI	Active, not recruiting	USA
NCT01811381	Phase 2	Longvida Curcumin® (800 mg/day of curcumin)	12 months	80 MCI	Recruiting	USA
NCT01504854	Phase 2	Resveratrol 500 mg/day with dose excalation by 500 mg increments ending with 2 g/day	52 weeks	119 Mild-to-moderate AD	Published	USA
NCT00743743	Phase 3	Longevinex brand resveratrol supplement (resveratrol 215 mg/day)	52 weeks	50 Mild-to-moderate AD	Withdrawn	USA
NCT00678431	Phase 3	Resveratrol with glucose and malate	12 months	27 Mild-to-moderate AD	Completed Result not available	USA
NCT01126229	Phase 1	Resveratrol 300 mg/day or 1,000 mg/day	12 weeks	32 ≥65 years old	Completed Result not available	USA
NCT01219244	Phase 4	Resveratrol or omega-3 supplementation or caloric restriction	6 months	330 MCI	Recruiting	Germany
NCT01766180	-	ResVida (resveratrol 150 mg/day) alone or associated with Fruitflow ^a^-II 150 mg/day	3 months	80 Subjects with memory impairment	Recruiting	USA
NCT02621554	Phase 2/Phase 3	Resveratrol (dose not reported)	12 months	60 ≥50 years Healthy or with subjective memory complaints	Recruiting	Germany
NCT02502253	Phase 1	Bioactive Dietary Polyphenol Preparation (BDPP) at low, moderate, and high dose	4 months	48 MCI	Recruiting	USA
NCT01982578	-	Genistein (60 mg BID)	180 days	50 AD	Unknown	Spain
NCT00205179	Phase 2	Soy isoflavones 100 mg/day; Novasoy® (85% daidzin and genistin, as glycosides)	6 month	59 AD	Published	USA
NCT00951834	Phase 2, 3	Epigallocatechin-gallate (EGCG) with increasing doses (in months 1–3: 200 mg EGCG, months 4–6: 400 mg, months 7–9: 600 mg and months 10–18: 800 mg EGCG).	18 month	21 subjects in the early stages of AD	Completed Results not available	Germany
NCT01699711	Phase 2	9 mg/kg of EGCG, (in green tea extract standardized for EGCG)	12 month	84 DS	Published	Spain
NCT01662414	Phase 4	Soy protein (as placebo) vs. HMS 90® (whey protein) by the dose of 1 sachect (10 g) 2 times/day as add-on (adjuvant) therapy.	6 month	32 Idiopathic PD	Published	Thailand
NCT02336633	–	Resveratrol (80 mg/j = 4 capsules/day)	12 month	120 HD	Recruiting	France
Not available	Pilot	Curcumin (600 mg/day, Brainoil) (for the first 3 months), followed by an open-label phase (for the last 3 months)	6 month	42ALS	Published	Italy
Not available	–	Nanocurcumin (80 mg daily)	12 month	54 Subjects with definite or probable ALS	Published	Iran

*AD, Alzheimer's disease; PD, Parkinson's disease; HD, Huntington disease; DS, Down syndrome; ALS, Amyotrophic Lateral Sclerosis*.

In a double-blinded, placebo-controlled trial in the United States, a mixture of curcumin derivatives (2 and 4 g/day), consisting of curcuminoids, demethoxycurcumin, and bisdemethoxycurcumin was prescribed to the patients with mild-to-moderate AD for 24 weeks and an open-label extension to 48 weeks. No significant differences in cognitive function and in plasma or cerebrospinal fluid (CSF) biomarkers of AD (including Aβ40 and Aβ42 levels, and total tau and p-tau) were observed between curcumin and the placebo groups (Ringman et al., 2012).

A double-blind, randomized, placebo-controlled trial in Iran was designed to evaluate the safety and efficacy of nanocurcumin in ALS adults. Subjects with a definite or probable ALS diagnosis were received either nanocurcumin (80 mg/day) or placebo for 12 months. The primary outcomes were considered to be survival/death and any mechanical ventilation dependency. The authors found a considerable difference between the study groups survival. No major adverse events or drug adverse effects and death were reported (Ahmadi et al., 2018).

Another pilot randomized trial was carried out in Italy to investigate the efficacy of the higher dose of oral curcumin (600 mg/day, Brainoil) on clinical parameters and biochemical markers, in 42 ALS patients. The first 3 months of the study was double blind and the last 3 months were open-label. Clinical criteria were consisted of ALS Functional Rating Scale Revised (ALS-FRS-r), BMI, Medical Research Council (MRC) scale, and Maximum Handgrip Force (MHF) scale; and the plasma metabolic biomarkers (i.e., plasma values of Advanced Oxidative Protein Products (AOPPs), ferric reducing ability (FRAP), total thiols (T-SH) groups and lactate). Data were not significant, however, the authors concluded further studies is required due to disparity of results (Chico et al., 2018).

#### Resveratrol

There are 5 recorded clinical trials related to the effects of resveratrol on AD (http://clinicaltrials. gov/ct2/results?term=alzheimerpmandpmresveratrol), 3 studies have been completed, 1 has been withdrawn and 1 is still active. Two clinical trials have reported that resveratrol altered several AD specific biomarkers with no major adverse effects in AD patients (Turner et al., 2015; Moussa et al., 2017). A phase 2 randomized, double-blind, placebo-controlled trial was performed in the United States, mainly to determine the safety, tolerability and pharmacokinetics of synthetic resveratrol (500 mg orally, once daily, increasing at 13 weeks intervals to a maximum of 1 g) in 119 individuals with mild to moderate AD for 52-weeks. They found that resveratrol was able to penetrate into the blood–brain barrier; likewise, the compound changed the AD biomarkers (Aβ40, Aβ42, tau, and phospho-tau) in the plasma and CSF. Plasma Aβ40 and CSF Aβ40 levels were found to be stabilized by resveratrol compared with a decrease in the placebo group; it also stabilized CSF Aβ42 in the subset of patients with biomarker-confirmed AD (baseline Aβ42 <600 ng/ml). Additionally, the brain volume loss was increased in resveratrol treated group (3 vs. 1%), probably due to anti-inflammatory activity of resveratrol. Totally, the results were not interpretable due to the study limitations; furthermore, no significant effects were found on clinical (secondary) outcomes. Resveratrol was found safe and well-tolerated with minor side effects such as nausea, diarrhea and weight loss (Turner et al., 2015).

Moussa et al., suggested that resveratrol treatment could preserve the integrity of the blood-brain barrier in AD patients with CSF Aβ42 <600 ng/ml, declined the level of CSF MMP9 (matrix metallopeptidase 9), and elevated macrophage-derived chemokine (MDC), interleukin (IL)-4, and the fibroblast growth factor (FGF)-2 (Moussa et al., 2017).

Moreover, resveratrol enhanced the plasma MMP10 and decreased the pro-inflammatory makers including IL-1R4, IL-12P40, IL-12P70, TNF-α, and RANTES compared to the baseline values. It also attenuated the accumulation of Aβs in the brain with no alterations in CSF tau and p-tau. From clinical point of view, resveratrol improved cognitive and functional decline (mini-mental state examination (MMSE) and activities of daily living) during 12-months study. The authors concluded that resveratrol reduced CSF MMP9, modified neuro-inflammatory factors, and caused adaptive immunity (Moussa et al., 2017).

Another randomized, double-blind phase 3 study has been carried out on 27 participants with mild-to-moderate AD. The treatment group received resveratrol (unknown dose) with glucose and malate, delivered in grape juice. Cognitive measurements (the ADAS-Cog scale and Clinical Global Impression of Change (CGIC) scale) were used at follow up visits at months 3, 6, 9, and 12 months. Although this study was completed, results are unpublished to date (Zhu et al., 2018).

One randomized double-blind placebo controlled trial is already running to investigate the effect of resveratrol on 102 early affected HD patients in France (5 ≤ UHDRS ≤ 40). Subjects received either resveratrol at 80 mg or placebo for 1 year. Clinical outcomes and biological tolerance evaluated every 3 months. The primary measure is the rate of caudate atrophy after 1-year treatment.

#### Genistein

Despite promising pre-clinical data, there is no clinical trials on the applications of genistein for AD treatment. Up to our knowledge, only one trial was conducted. Genistein (60 mg) administration for 180 days changed the Aβ level in CSF of AD patients, besides, improved MMSE, ADAS-cog and the memory alteration test at determined intervals. Although, the study has passed its completion date, but the results are not available (Yassa et al., 2009).

#### Soy Isoflavones

There is a pilot randomized clinical trial examining the effect of soy isoflavones on cognitive function in old men and women over the age of 60 diagnosed with AD. Sixty-five participants were randomized to treatment for 6 months by soy isoflavone (100 mg/day; 85% daidzin and genistin as glycosides), or matching placebo capsules. No significant differences were observed between isoflavones treated group and placebo group or between the genders in terms of cognition outcomes, and self-report of mood symptoms. Besides, it was found that cognitive functions (speed dexterity and verbal fluency) were associated with equal levels (Gleason et al., 2015). A double-blind, placebo-controlled, Phase IV trial was designed in Thailand to compare HMS 90® (an immune system stimulant) vs. soy protein (as placebo), by the dose of 1 sachet (10 g) 2 times/day as adjuvant therapy in 38 individuals with idiopathic PD. No significant clinical outcomes were observed in biomarkers of oxidative stress (glutathione), plasma amino acids, and the brain function in both groups (Tosukhowong et al., 2016).

#### Epigallocatechin Gallate

The normalization of tyrosine phosphorylation regulated kinase 1A gene (Dyrk1A) and APP functions as therapeutic approaches for cognition improvement and slowing AD progression was investigated in a phase 2 randomized clinical study. Down syndrome (DS) patients received a daily oral dose of 9 mg/kg of EGCG, (in green tea extract standardized for EGCG) for 12 months. EGCG caused significant improvement in the adaptive behavior and brain-related changes in young adults with DS (de la Torre et al., 2016). In a phase 2 randomized placebo-controlled clinical trial, 21 patients over the age of 60 and in the early stages of AD received EGCG in an increasing manner (in months 1–3: 200 mg EGCG, months 4–6: 400 mg, months 7–9: 600 mg, and months 10–18: 800 mg EGCG). ADAS-COG score, MMSE score, safety and tolerability, and the brain atrophy were assessed, although the study is completed but data are not available (https://clinicaltrials.gov/ct2/show/NCT00951834).

## Conclusion and Future Prospects

The unique involvement of UPS in malfunction of the nervous system encloses a broad range from drug abuse to neuroinflammation, making UPS an emerging topic in neurodegeneration and an important target for drug discovery. Since, UPS function is downregulated in vulnerable degenerating neurons in neurodegenerative diseases, thus, normal function of UPS assures a balanced regulation of misfolded protein degradation, which contributes to eliminate abnormal protein aggregates and to maintain the cellular protein homeostasis in the brain and neural network.

So far, a number of UPS regulators were developed. For example, cAMP phosphodiesterases inhibitors and UCHL1 that can modulate the brain cAMP-dependent protein kinase A (PKA)-cAMP response element binding protein (CREB) PKA-pCREB levels in AD subjects, resulting in enhanced protein degradation and synaptic functions (Cao et al., 2019). According to the collected data, a number of PPs are able to exert UPS inhibitory activity, mainly through chymotrypsin-like activity on both intracellular 26S and purified 20S proteasome. PPs interfere in many steps of degradation processes by means of proteasome inhibition, deubiquitinase activity, and stimulation of E1, E2, or E3, resulting in reduction of neurotoxicity, improvement of synaptic plasticity, and transmission, as well as enhanced neuronal survival. In this context, few clinical trials were carried out, mostly on a limited basis, however, the results are inconclusive and in most cases statistically insignificant. However, concerning PPs and their probable interaction with UPS, two hypotheses can be speculated; either their proteasomal inhibitory effects or their proteasomal stimulatory functions. PPs can induce ubiquitination which results in accelerating the elimination of damaged soluble proteins and degradation of short-lived regulatory proteins. Another way, inhibition of proteasomal activity by proteasome inhibitors i.e., PPs has been linked to synaptic plasticity.

Considering PPs and their roles in neuroprotection, curcumin, and resveratrol are the most studied polyphenolic compounds followed by EGCG and genistein, alone or in combination with other conventional drugs. Recently, it was proposed that UPS dysregulation, aberrant mRNA splicing, mitochondrial dysfunction, and excessive oxidative stress directly interplay with the process of neurodegeneration, thereby, future design of biomarkers and the drug development plans have to focus on concurrent targeting of multiple components and steps of neurodegenerative diseases (Tan et al., 2019). It is plausible that a combination of PPs and current drugs may improve the PPs therapeutic application for neuronal related destruction disorders. In this way, PPs offers considerable opportunity for development of specific therapeutics approaches via UPS, for particular groups of misfolded proteins. However, direct links and molecular mechanisms remain elusive and require to be addressed.

This is important to have a clear understanding of PPs molecular mechanism of action and their possible interplay with UPS. In addition, the upstream regulators or downstream targets of UPS have to be characterized, empowering researches to develop treatment strategies with more specificity and efficacy. Another question that has to be responded is whether the inhibition of UPS by PPs is more favorable for the neurodegenerative diseases or the stimulation of this system? Concerning neurodegeneration, further clinical interventions with greater sample size, proper duration, applicable formulations, and/or dosages should be designed to assess the potential efficacy of natural bioactive compounds as UPS inhibitors/regulators.

## Author Contributions

ZM, FE-S, NS, FG, MK, and AA contributed to the collection and/or assembly of data and interpretation, and the writing of the manuscript. SM, MA, and MF contributed to the provision of study material, conception and design, and final approval of manuscript. All authors have read and approved the manuscript.

## Conflict of Interest

The authors declare that the research was conducted in the absence of any commercial or financial relationships that could be construed as a potential conflict of interest.

## References

[B1] Ahmadi M. Agah E. Nafissi S. Jaafari M. R. Harirchian M. H. Sarraf P.. (2018). Safety and efficacy of nanocurcumin as add-on therapy to riluzole in patients with amyotrophic lateral sclerosis: a pilot randomized clinical trial. Neurotherapeutics 15, 430–438. 10.1007/s13311-018-0606-7 29352425 PMC5935637

[B2] Ahtoniemi T. Goldsteins G. Keksa-Goldsteine V. Malm T. Kanninen K. Salminen A.. (2007). Pyrrolidine dithiocarbamate inhibits induction of immunoproteasome and decreases survival in a rat model of amyotrophic lateral sclerosis. Mol. Pharmacol. 71, 30–37. 10.1124/mol.106.028415 17008387

[B3] Aizawa M. Abe Y. Ito T. Handa H. Nawa H. (2011). mRNA distribution of the thalidomide binding protein cereblon in adult mouse brain. Neurosci. Res. 69, 343–347. 10.1016/j.neures.2010.12.019 21241746

[B4] Akutsu M. Dikic I. Bremm A. (2016). Ubiquitin chain diversity at a glance. J. Cell Sci. 129, 875–880. 10.1242/jcs.183954 26906419

[B5] Almeida C. G. Takahashi R. H. Gouras G. K. (2006). Beta-amyloid accumulation impairs multivesicular body sorting by inhibiting the ubiquitin-proteasome system. J. Neurosci. 26, 4277–4288. 10.1523/JNEUROSCI.5078-05.2006 16624948 PMC6673997

[B6] Al-Shami A. Jhaver K. G. Vogel P. Wilkins C. Humphries J. Davis J. J.. (2010). Regulators of the proteasome pathway, Uch37 and Rpn13, play distinct roles in mouse development. PLoS ONE 5:e13654. 10.1371/journal.pone.0013654 21048919 PMC2965108

[B7] Attal M. Harousseau J.-L. Stoppa A.-M. Sotto J.-J. Fuzibet J.-G. Rossi J.-F.. (1996). A prospective, randomized trial of autologous bone marrow transplantation and chemotherapy in multiple myeloma. N. Engl. J. Med. 335, 91–97. 10.1056/NEJM199607113350204 8649495

[B8] Ayissi V. B. Ebrahimi A. Schluesenner H. (2014). Epigenetic effects of natural polyphenols: a focus on SIRT1-mediated mechanisms. Mol. Nutr. Food Res. 58, 22–32. 10.1002/mnfr.201300195 23881751

[B9] Babitha K. Shanmuga Sundaram R. Annapandian V. Abhirama B. Sudha M. Thiyagarajan T. (2014). Natural products and its derived drugs for the treatment of neurodegenerative disorders: Alzheimer's disease–a review. Br. Biomed. Bull. 2, 359–370.

[B10] Bagli E. Goussia A. Moschos M. M. Agnantis N. Kitsos G. (2016). Natural compounds and neuroprotection: mechanisms of action and novel delivery systems. In Vivo 30, 535–547. 27566070

[B11] Banerjee K. Munshi S. Sen O. Pramanik V. Roy Mukherjee T. Chakrabarti S. (2014). Dopamine cytotoxicity involves both oxidative and nonoxidative pathways in SH-SY5Y cells: potential role of alpha-synuclein overexpression and proteasomal inhibition in the etiopathogenesis of Parkinson's disease. Parkinsons Dis. 2014:878935. 10.1155/2014/878935 24804146 PMC3996320

[B12] Baum L. Lam C. W. Cheung S. K. Kwok T. Lui V. Tsoh J.. (2008). Six-month randomized, placebo-controlled, double-blind, pilot clinical trial of curcumin in patients with Alzheimer disease. J. Clin. Psychopharmacol. 28, 110–113. 10.1097/jcp.0b013e318160862c 18204357

[B13] Bendall H. H. Sikes M. L. Ballard D. W. Oltz E. M. (1999). An intact NF-κB signaling pathway is required for maintenance of mature B cell subsets. Mol. Immunol. 36, 187–195. 10.1016/s0161-5890(99)00031-0 10403484

[B14] Bendotti C. Marino M. Cheroni C. Fontana E. Crippa V. Poletti A.. (2012). Dysfunction of constitutive and inducible ubiquitin-proteasome system in amyotrophic lateral sclerosis: implication for protein aggregation and immune response. Prog. Neurobiol. 97, 101–126. 10.1016/j.pneurobio.2011.10.001 22033150

[B15] Bennett E. J. Shaler T. A. Woodman B. Ryu K.-Y. Zaitseva T. S. Becker C. H.. (2007). Global changes to the ubiquitin system in Huntington's disease. Nature 448:704. 10.1038/nature06022 17687326

[B16] Bilguvar K. Tyagi N. K. Ozkara C. Tuysuz B. Bakircioglu M. Choi M.. (2013). Recessive loss of function of the neuronal ubiquitin hydrolase UCHL1 leads to early-onset progressive neurodegeneration. Proc. Natl. Acad. Sci. U.S.A. 110, 3489–3494. 10.1073/pnas.1222732110 23359680 PMC3587195

[B17] Blackinton J. G. Anvret A. Beilina A. Olson L. Cookson M. R. Galter D. (2007). Expression of PINK1 mRNA in human and rodent brain and in Parkinson's disease. Brain. Res. 1184, 10–16. 10.1016/j.brainres.2007.09.056 17950257

[B18] Borodovsky A. Kessler B. M. Casagrande R. Overkleeft H. S. Wilkinson K. D. Ploegh H. L. (2001). A novel active site-directed probe specific for deubiquitylating enzymes reveals proteasome association of USP14. EMBO J. 20, 5187–5196. 10.1093/emboj/20.18.5187 11566882 PMC125629

[B19] Bosco D. A. Morfini G. Karabacak N. M. Song Y. Gros-Louis F. Pasinelli P.. (2010). Wild-type and mutant SOD1 share an aberrant conformation and a common pathogenic pathway in ALS. Nat. Neurosci. 13:1396. 10.1038/nn.2660 20953194 PMC2967729

[B20] Braidy N. Jugder B.-E. Poljak A. Jayasena T. Mansour H. Mohammad Nabavi S.. (2016). Resveratrol as a potential therapeutic candidate for the treatment and management of Alzheimer's disease. Curr. Top Med. Chem. 16, 1951–1960. 10.2174/1568026616666160204121431 26845555

[B21] Branquinho Andrade P. Grosso C. Valentao P. Bernardo J. (2016). Flavonoids in neurodegeneration: limitations and strategies to cross CNS barriers. Curr. Med. Chem. 23, 4151–4174. 10.2174/0929867323666160809094934 27516197

[B22] Brewer J. W. Diehl J. A. (2000). PERK mediates cell-cycle exit during the mammalian unfolded protein response. Proc. Natl. Acad. Sci. U.S.A. 97, 12625–12630. 10.1073/pnas.220247197 11035797 PMC18814

[B23] Briggs R. Kennelly S. P. O'neill D. (2016). Drug treatments in Alzheimer's disease. Clin. Med. 16, 247–253. 10.7861/clinmedicine.16-3-247 27251914 PMC5922703

[B24] Brockman J. A. Scherer D. C. McKinsey T. A. Hall S. M. Qi X. Lee W. Y.. (1995). Coupling of a signal response domain in I kappa B alpha to multiple pathways for NF-kappa B activation. Mol. Cell Biol. 15, 2809–2818. 10.1128/mcb.15.5.2809 7739562 PMC230512

[B25] Budd Haeberlein S. O'Gorman J. Chiao P. Bussiere T. von Rosenstiel P. Tian Y.. (2017). Clinical development of aducanumab, an anti-abeta human monoclonal antibody being investigated for the treatment of early Alzheimer's disease. J. Prev. Alzheimers Dis. 4, 255–263. 10.14283/jpad.2017.39 29181491

[B26] Cai Z. Zeng W. Tao K. Lu F. Gao G. Yang Q. (2015). Myricitrin alleviates MPP(+)-induced mitochondrial dysfunction in a DJ-1-dependent manner in SN4741 cells. Biochem. Biophys. Res. Commun. 458, 227–233. 10.1016/j.bbrc.2015.01.060 25623535

[B27] Cao J. Zhong M. B. Toro C. A. Zhang L. Cai D. (2019). Endo-lysosomal pathway and ubiquitin-proteasome system dysfunction in Alzheimer's disease pathogenesis. Neurosci. Lett. 703, 68–78. 10.1016/j.neulet.2019.03.016 30890471 PMC6760990

[B28] Cao K. Zheng A. Xu J. Li H. Liu J. Peng Y.. (2014). AMPK activation prevents prenatal stress-induced cognitive impairment: modulation of mitochondrial content and oxidative stress. Free Radic. Biol. Med. 75, 156–166. 10.1016/j.freeradbiomed.2014.07.029 25091899

[B29] Cecarini V. Bonfili L. Cuccioloni M. Mozzicafreddo M. Rossi G. Keller J. N.. (2014). Wild type and mutant amyloid precursor proteins influence downstream effects of proteasome and autophagy inhibition. Biochim. Biophys. Acta 1842, 127–134. 10.1016/j.bbadis.2013.11.002 24215712

[B30] Chang T.-L. Chiang H.-Y. Shen J.-Y. Lin S.-W. Tsai P.-J. (2015). Phenolic compounds stage an interplay between the ubiquitin–proteasome system and ubiquitin signal autophagic degradation for the ubiquitin-based cancer chemoprevention. J. Funct. Food 17, 857–871. 10.1016/j.jff.2015.06.010

[B31] Chauhan D. Li G. Hideshima T. Podar K. Mitsiades C. Mitsiades N.. (2003). JNK-dependent release of mitochondrial protein, Smac, during apoptosis in multiple myeloma (MM) cells. J. Biol. Chem. 278, 17593–17596. 10.1074/jbc.C300076200 12665525

[B32] Chen D. Chen M. S. Cui Q. C. Yang H. Dou Q. P. (2007a). Structure-proteasome-inhibitory activity relationships of dietary flavonoids in human cancer cells. Front. Biosci. 12, 1935–1945. 10.2741/2199 17127432

[B33] Chen D. Landis-Piwowar K. R. Chen M. S. Dou Q. P. (2007b). Inhibition of proteasome activity by the dietary flavonoid apigenin is associated with growth inhibition in cultured breast cancer cells and xenografts. Breast Cancer Res. 9:R80. 10.1186/bcr1797 18300387 PMC2246179

[B34] Chen J. J. Ondo W. G. Dashtipour K. Swope D. M. (2012). Tetrabenazine for the treatment of hyperkinetic movement disorders: a review of the literature. Clin. Ther. 34, 1487–1504. 10.1016/j.clinthera.2012.06.010 22749259

[B35] Chen L. Thiruchelvam M. J. Madura K. Richfield E. K. (2006). Proteasome dysfunction in aged human α-synuclein transgenic mice. Neurobiol. Dis. 23, 120–126. 10.1016/j.nbd.2006.02.004 16713278

[B36] Cheroni C. Marino M. Tortarolo M. Veglianese P. de Biasi S. Fontana E.. (2008). Functional alterations of the ubiquitin-proteasome system in motor neurons of a mouse model of familial amyotrophic lateral sclerosis. Hum. Mol. Genet. 18, 82–96. 10.1093/hmg/ddn319 18826962 PMC3298865

[B37] Chico L. Ienco E. C. Bisordi C. Lo Gerfo A. Petrozzi L. Petrucci A.. (2018). Amyotrophic lateral sclerosis and oxidative stress: a double-blind therapeutic trial after curcumin supplementation. CNS Neurol. Disord. Drug Targets 17, 767–779. 10.2174/1871527317666180720162029 30033879

[B38] Choudhary S. Kumar P. Malik J. (2013). Plants and phytochemicals for Huntington's disease. Pharmacogn. Rev. 7, 81–91. 10.4103/0973-7847.120505 24347915 PMC3841999

[B39] Ciechanover A. (2005). Proteolysis: from the lysosome to ubiquitin and the proteasome. Nat. Rev. Mol. Cell Biol. 6:79. 10.1038/nrm1552 15688069

[B40] Clements C. M. McNally R. S. Conti B. J. Mak T. W. Ting J. P.-Y. (2006). DJ-1, a cancer-and Parkinson's disease-associated protein, stabilizes the antioxidant transcriptional master regulator Nrf2. Proc. Natl. Acad. Sci. U.S.A. 103, 15091–15096. 10.1073/pnas.0607260103 17015834 PMC1586179

[B41] Cookson M. R. Bandmann O. (2010). Parkinson's disease: insights from pathways. Hum. Mol. Genet. 19, R21–R27. 10.1093/hmg/ddq167 20421364 PMC2875048

[B42] Crippa V. Sau D. Rusmini P. Boncoraglio A. Onesto E. Bolzoni E.. (2010). The small heat shock protein B8 (HspB8) promotes autophagic removal of misfolded proteins involved in amyotrophic lateral sclerosis (ALS). Hum. Mol. Genet. 19, 3440–3456. 10.1093/hmg/ddq257 20570967

[B43] Cummins J. M. Rago C. Kohli M. Kinzler K. W. Lengauer C. Vogelstein B. (2004). Tumour suppression: disruption of HAUSP gene stabilizes. Nature 428, 1–2. 10.1038/nature02501 15058298

[B44] D'Angiolella V. Esencay M. Pagano M. (2013). A cyclin without cyclin-dependent kinases: cyclin F controls genome stability through ubiquitin-mediated proteolysis. Trends Cell Biol. 23, 135–140. 10.1016/j.tcb.2012.10.011 23182110 PMC3597434

[B45] Davinelli S. Sapere N. Visentin M. Zella D. Scapagnini G. (2013). Enhancement of mitochondrial biogenesis with polyphenols: combined effects of resveratrol and equol in human endothelial cells. Immun. Ageing 10:28. 10.1186/1742-4933-10-28 23842073 PMC3750512

[B46] de la Torre R. de Sola S. Hernandez G. Farre M. Pujol J. Rodriguez J.. (2016). Safety and efficacy of cognitive training plus epigallocatechin-3-gallate in young adults with Down's syndrome (TESDAD): a double-blind, randomised, placebo-controlled, phase 2 trial. Lancet Neurol. 15, 801–810. 10.1016/S1474-4422(16)30034-5 27302362

[B47] de Oliveira M. R. Nabavi S. F. Habtemariam S. Erdogan Orhan I. Daglia M. Nabavi S. M. (2015). The effects of baicalein and baicalin on mitochondrial function and dynamics: a review. Pharmacol. Res. 100, 296–308. 10.1016/j.phrs.2015.08.021 26318266

[B48] de Oliveira M. R. Nabavi S. F. Manayi A. Daglia M. Hajheydari Z. Nabavi S. M. (2016). Resveratrol and the mitochondria: From triggering the intrinsic apoptotic pathway to inducing mitochondrial biogenesis, a mechanistic view. Biochim. Biophys. Acta 1860, 727–745. 10.1016/j.bbagen.2016.01.017 26802309

[B49] de Rus Jacquet A. Tambe M. A. Rochet J. C. (2017a). Dietary phytochemicals in neurodegenerative disease, in Nutrition in the Prevention and Treatment of Disease, 4th ed eds Coulston A. M. Boushey C. J. Ferruzzi M. G. Delahanty L. M. (Elsevier; Academic Press), 361–391. 10.1016/B978-0-12-802928-2.00018-7

[B50] de Rus Jacquet A. Timmers M. Ma S. Y. Thieme A. McCabe G. P. Vest J. H. C.. (2017b). Lumbee traditional medicine: neuroprotective activities of medicinal plants used to treat Parkinson's disease-related symptoms. J. Ethnopharmacol. 206, 408–425. 10.1016/j.jep.2017.02.021 28214539 PMC6149226

[B51] del Rosario Campos-Esparza M. Adriana Torres-Ramos M. (2010). Neuroprotection by natural polyphenols: molecular mechanisms. Cent. Nerv. Syst. Agents Med. Chem. 10, 269–277. 10.2174/187152410793429728 20868360

[B52] Deng H.-X. Chen W. Hong S.-T. Boycott K. M. Gorrie G. H. Siddique N.. (2011). Mutations in UBQLN2 cause dominant X-linked juvenile and adult-onset ALS and ALS/dementia. Nature 477, 211–215. 10.1038/nature10353 21857683 PMC3169705

[B53] Deng H. X. Zhai H. Bigio E. H. Yan J. Fecto F. Ajroud K.. (2010). FUS-immunoreactive inclusions are a common feature in sporadic and non-SOD1 familial amyotrophic lateral sclerosis. Ann. Neurol. 67, 739–748. 10.1002/ana.22051 20517935 PMC4376270

[B54] Dennissen F. Kholod N. van Leeuwen F. (2012). The ubiquitin proteasome system in neurodegenerative diseases: culprit, accomplice or victim? Prog. Neurobiol. 96, 190–207. 10.1016/j.pneurobio.2012.01.003 22270043

[B55] Devi K. P. Shanmuganathan B. Manayi A. Nabavi S. F. Nabavi S. M. (2017). Molecular and therapeutic targets of genistein in Alzheimer's disease. Mol. Neurobiol. 54, 7028–7041. 10.1007/s12035-016-0215-6 27796744

[B56] Dey A. De J. N. (2015). Neuroprotective therapeutics from botanicals and phytochemicals against Huntington's disease and related neurodegenerative disorders. J. Herb. Med. 5, 1–19. 10.1016/j.hermed.2015.01.002

[B57] DiFiglia M. Sapp E. Chase K. O. Davies S. W. Bates G. P. Vonsattel J.. (1997). Aggregation of huntingtin in neuronal intranuclear inclusions and dystrophic neurites in brain. Science 277, 1990–1993. 10.1126/science.277.5334.1990 9302293

[B58] Dil Kuazi A. Kito K. Abe Y. Shin R. W. Kamitani T. Ueda N. (2003). NEDD8 protein is involved in ubiquitinated inclusion bodies. J. Pathol. 199, 259–266. 10.1002/path.1283 12533840

[B59] Ding Y. Chen X. Wang B. Yu B. Ge J. Shi X. (2018). Quercetin suppresses the chymotrypsin-like activity of proteasome via inhibition of MEK1/ERK1/2 signaling pathway in hepatocellular carcinoma HepG2 cells. Can. J. Physiol. Pharmacol. 96, 521–526. 10.1139/cjpp-2017-0655 29394494

[B60] Dolan P. J. Johnson G. V. (2010). A caspase cleaved form of tau is preferentially degraded through the autophagy pathway. J. Biol. Chem. 110:110940 10.1074/jbc.M110.110940 PMC2903354 20466727

[B61] Dreiseitel A. Schreier P. Oehme A. Locher S. Rogler G. Piberger H.. (2008). Inhibition of proteasome activity by anthocyanins and anthocyanidins. Biochem. Biophys. Res. Commun. 372, 57–61. 10.1016/j.bbrc.2008.04.140 18460339

[B62] Ellgaard L. Molinari M. Helenius A. (1999). Setting the standards: quality control in the secretory pathway. Science 286, 1882–1888. 10.1126/science.286.5446.1882 10583943

[B63] Esteras N. Muñoz Ú. Alquézar C. Bartolomé F. Bermejo-Pareja F. Martín-Requero Á. (2012). Altered calmodulin degradation and signaling in non-neuronal cells from Alzheimer's disease patients. Curr. Alzheimers Res. 9, 267–277. 10.2174/156720512800107564 22044025

[B64] Everett R. D. Meredith M. Orr A. Cross A. Kathoria M. Parkinson J. (1997). A novel ubiquitin-specific protease is dynamically associated with the PML nuclear domain and binds to a herpesvirus regulatory protein. EMBO J. 16, 566–577. 10.1093/emboj/16.3.566 9034339 PMC1169660

[B65] Farooqui T. Farooqui A. A. (2018). Neuroprotective effects of garlic in model systems of neurodegenerative diseases, in Role of the Mediterranean Diet in the Brain and Neurodegenerative Diseases, eds Farooqui T. Farooqui A. A. (Elsevier; Academic Press), 253–269. 10.1016/B978-0-12-811959-4.00016-X

[B66] Farzaei M. H. Shahpiri Z. Mehri M. R. Bahramsoltani R. Rezaei M. Raeesdana A.. (2018a). Medicinal plants in neurodegenerative diseases: perspective of traditional persian medicine. Curr. Drug Metabol. 19, 429–442. 10.2174/1389200219666180305150256 29512453

[B67] Farzaei M. H. Tewari D. Momtaz S. Argüelles S. Nabavi S. M. (2018b). Targeting ERK signaling pathway by polyphenols as novel therapeutic strategy for neurodegeneration. Food Chem. Toxicol. 120, 183–195. 10.1016/j.fct.2018.07.010 29981370

[B68] Filipits M. Pohl G. Stranzl T. Kaufmann H. Ackermann J. Gisslinger H.. (2003). Low p27Kip1 expression is an independent adverse prognostic factor in patients with multiple myeloma. Clin. Cancer Res. 9, 820–826. 12576455

[B69] Fiorani M. Guidarelli A. Blasa M. Azzolini C. Candiracci M. Piatti E.. (2010). Mitochondria accumulate large amounts of quercetin: prevention of mitochondrial damage and release upon oxidation of the extramitochondrial fraction of the flavonoid. J. Nutr. Biochem. 21, 397–404. 10.1016/j.jnutbio.2009.01.014 19278846

[B70] Fischer E. S. Böhm K. Lydeard J. R. Yang H. Stadler M. B. Cavadini S.. (2014). Structure of the DDB1-CRBN E3 ubiquitin ligase in complex with thalidomide. Nature 512, 49. 10.1038/nature13527 25043012 PMC4423819

[B71] Freemont P. S. (2000). Ubiquitination: RING for destruction? Curr. Biol. 10, R84–R87. 10.1016/S0960-9822(00)00287-6 10662664

[B72] Giacoppo S. Galuppo M. Montaut S. Iori R. Rollin P. Bramanti P.. (2015). An overview on neuroprotective effects of isothiocyanates for the treatment of neurodegenerative diseases. Fitoterapia 106, 12–21. 10.1016/j.fitote.2015.08.001 26254971

[B73] Giguere N. Pacelli C. Saumure C. Bourque M.-J. Matheoud D. Levesque D. (2018). Comparative analysis of Parkinson's disease–associated genes reveals altered survival and bioenergetics of parkin-deficient dopamine neurons in mice. J. Biol. Chem. 117:000499 10.1074/jbc.RA117.000499 PMC6016451 29700116

[B74] Gleason C. E. Fischer B. L. Dowling N. M. Setchell K. D. R. Atwood C. S. Carlsson C. M.. (2015). Cognitive effects of soy isoflavones in patients with Alzheimer's disease. J. Alzheimers Dis. 47, 1009–1019. 10.3233/JAD-142958 26401779 PMC4657545

[B75] Gong B. Cao Z. Zheng P. Vitolo O. V. Liu S. Staniszewski A.. (2006). Ubiquitin hydrolase Uch-L1 rescues β-amyloid-induced decreases in synaptic function and contextual memory. Cell 126, 775–788. 10.1016/j.cell.2006.06.046 16923396

[B76] Gorji N. Moeini R. Memariani Z. (2018). Almond, hazelnut and walnut, three nuts for neuroprotection in Alzheimer's disease: a neuropharmacological review of their bioactive constituents. Pharmacol. Res. 129, 115–127. 10.1016/j.phrs.2017.12.003 29208493

[B77] Grice G. L. Nathan J. A. (2016). The recognition of ubiquitinated proteins by the proteasome. Cell Mol. Life Sci. 73, 3497–3506. 10.1007/s00018-016-2255-5 27137187 PMC4980412

[B78] Grune T. Botzen D. Engels M. Voss P. Kaiser B. Jung T.. (2010). Tau protein degradation is catalyzed by the ATP/ubiquitin-independent 20S proteasome under normal cell conditions. Arch. Biochem. Biophys. 500, 181–188. 10.1016/j.abb.2010.05.008 20478262 PMC2904402

[B79] Gu P. S. Moon M. Choi J. G. Oh M. S. (2017). Mulberry fruit ameliorates Parkinson's-disease-related pathology by reducing α-synuclein and ubiquitin levels in a 1-methyl-4-phenyl-1, 2, 3, 6-tetrahydropyridine/probenecid model. J. Nutr. Biochem. 39, 15–21. 10.1016/j.jnutbio.2016.08.014 27741433

[B80] Haass C. Selkoe D. J. (2007). Soluble protein oligomers in neurodegeneration: lessons from the Alzheimer's amyloid β-peptide. Nat. Rev. Mol. Cell Biol. 8:101. 10.1038/nrm2101 17245412

[B81] Hajieva P. (2017). The effect of polyphenols on protein degradation pathways: implications for neuroprotection. Molecules 22:159. 10.3390/molecules22010159 28106854 PMC6155800

[B82] Hardiman O. van den Berg L. H. Kiernan M. C. (2011). Clinical diagnosis and management of amyotrophic lateral sclerosis. Nat. Rev. Neurol. 7:639. 10.1038/nrneurol.2011.153 21989247

[B83] Hasan W. Kori R. K. Jain J. Yadav R. S. Jat D. (2019b). Neuroprotective effects of mitochondria-targeted curcumin against rotenone-induced oxidative damage in cerebellum of mice. J. Biochem. Mol. Toxicol. 34:e22416. 10.1002/jbt.22416 31714633

[B84] Hasan W. Kori R. K. Thakre K. Yadav R. S. Jat D. (2019a). Synthesis, characterization and efficacy of mitochondrial targeted delivery of TPP-curcumin in rotenone-induced toxicity. DARU J. Pharm. Sci. 27, 557–570. 10.1007/s40199-019-00283-2 31264184 PMC6895339

[B85] Heeman B. van den Haute C. Aelvoet S. -A. Valsecchi F. Rodenburg R. J. Reumers V.. (2011). Depletion of PINK1 affects mitochondrial metabolism, calcium homeostasis and energy maintenance. J. Cell Sci. 124, 1115–1125. 10.1242/jcs.078303. 21385841

[B86] Hegde A. N. van Leeuwen F. W. (2017). Ubiquitin and the brain: roles of proteolysis in the normal and abnormal nervous system. Front. Mol. Neurosci. 10:220. 10.3389/fnmol.2017.00220 28769757 PMC5513962

[B87] Hernandez F. Avila J. (2007). Tauopathies. Cell Mol. Life Sci. 64, 2219–2233. 10.1007/s00018-007-7220-x 17604998 PMC11136052

[B88] Hideshima T. Richardson P. Chauhan D. Palombella V. J. Elliott P. J. Adams J.. (2001). The proteasome inhibitor PS-341 inhibits growth, induces apoptosis, and overcomes drug resistance in human multiple myeloma cells. Cancer Res. 61, 3071–3076. 11306489

[B89] Higgins J. J. Hao J. Kosofsky B. E. Rajadhyaksha A. M. (2008). Dysregulation of large-conductance Ca 2+-activated K+ channel expression in nonsyndromal mental retardation due to a cereblon p. R419X mutation. Neurogenetics 9, 219–223. 10.1007/s10048-008-0128-2 18414909

[B90] Higgins J. J. Tal A. L. Sun X. Hauck S. C. Hao J. Kosofosky B. E.. (2010). Temporal and spatial mouse brain expression of cereblon, an ionic channel regulator involved in human intelligence. J. Neurogenet. 24, 18–26. 10.3109/01677060903567849 20131966

[B91] Hiramatsu Y. Kitagawa K. Suzuki T. Uchida C. Hattori T. Kikuchi H.. (2006). Degradation of Tob1 mediated by SCFSkp2-dependent ubiquitination. Cancer Res. 66, 8477–8483. 10.1158/0008-5472.CAN-06-1603 16951159

[B92] Hohberger B. Enz R. (2009). Cereblon is expressed in the retina and binds to voltage-gated chloride channels. FEBS Lett. 583, 633–637. 10.1016/j.febslet.2009.01.018 19166841

[B93] Houde C. Li Y. Song L. Barton K. Zhang Q. Godwin J.. (2004). Overexpression of the NOTCH ligand JAG2 in malignant plasma cells from multiple myeloma patients and cell lines. Blood 104, 3697–3704. 10.1182/blood-2003-12-4114 15292061

[B94] Hu M. Li P. Li M. Li W. Yao T. Wu J.-W.. (2002). Crystal structure of a UBP-family deubiquitinating enzyme in isolation and in complex with ubiquitin aldehyde. Cell 111, 1041–1054. 10.1016/s0092-8674(02)01199-6 12507430

[B95] Hu S. Maiti P. Ma Q. Zuo X. Jones M. R. Cole G. M.. (2015). Clinical development of curcumin in neurodegenerative disease. Expert. Rev. Neurother. 15, 629–637. 10.1586/14737175.2015.1044981 26035622 PMC6800094

[B96] Huang H. Regan K. M. Wang F. Wang D. Smith D. I. van Deursen J. M.. (2005). Skp2 inhibits FOXO1 in tumor suppression through ubiquitin-mediated degradation. Proc. Natl. Acad. Sci. U.S.A. 102, 1649–1654. 10.1073/pnas.0406789102 15668399 PMC545492

[B97] Hyttinen J. M. Amadio M. Viiri J. Pascale A. Salminen A. Kaarniranta K. (2014). Clearance of misfolded and aggregated proteins by aggrephagy and implications for aggregation diseases. Ageing Res. Rev. 18, 16–28. 10.1016/j.arr.2014.07.002 25062811

[B98] Ibba M. Söll D. (1999). Quality control mechanisms during translation. Science 286, 1893–1897. 10.1126/science.286.5446.1893 10583945

[B99] Ip P. Sharda P. R. Cunningham A. Chakrabartty S. Pande V. Chakrabartty A. (2017). Quercitrin and quercetin 3-β-d-glucoside as chemical chaperones for the A4V SOD1 ALS-causing mutant. Protein Eng. Des. Sel. 30, 431–440. 10.1093/protein/gzx025 28475686 PMC5939853

[B100] Ito T. Ando H. Suzuki T. Ogura T. Hotta K. Imamura Y.. (2010). Identification of a primary target of thalidomide teratogenicity. Science 327, 1345–1350. 10.1126/science.1177319 20223979

[B101] Jaiswal M. K. (2018). Riluzole and edaravone: a tale of two amyotrophic lateral sclerosis drugs. Med. Res. Rev. 39, 733–748. 10.1002/med.21528 30101496

[B102] Jakob C. Egerer K. Liebisch P. Türkmen S. Zavrski I. Kuckelkorn U.. (2007). Circulating proteasome levels are an independent prognostic factor for survival in multiple myeloma. Blood 109, 2100–2105. 10.1182/blood-2006-04-016360 17095627

[B103] Jana N. R. Dikshit P. Goswami A. Nukina N. (2004). Inhibition of proteasomal function by curcumin induces apoptosis through mitochondrial pathway. J. Biol. Chem. 279, 11680–11685. 10.1074/jbc.M310369200 14701837

[B104] Jankovic J. Poewe W. (2012). Therapies in Parkinson's disease. Curr. Opin. Neurol. 25, 433–447. 10.1097/WCO.0b013e3283542fc2 22691758

[B105] Jara J. H. Frank D. D. Özdinler P.H. (2013). Could dysregulation of UPS be a common underlying mechanism for cancer and neurodegeneration? Lessons from UCHL1. Cell Biochem. Biophys. 67, 45–53. 10.1007/s12013-013-9631-7 23695785

[B106] Jo S. Lee K. H. Song S. Jung Y. K. Park C. S. (2005). Identification and functional characterization of cereblon as a binding protein for large-conductance calcium-activated potassium channel in rat brain. J. Neurochem. 94, 1212–1224. 10.1111/j.1471-4159.2005.03344.x 16045448

[B107] Johnson J. O. Mandrioli J. Benatar M. Abramzon Y. van Deerlin V. M. Trojanowski J. Q.. (2010). Exome sequencing reveals VCP mutations as a cause of familial ALS. Neuron 68, 857–864. 10.1016/j.neuron.2010.11.036 21145000 PMC3032425

[B108] Joshi V. Mishra R. Upadhyay A. Amanullah A. Poluri K. M. Singh S.. (2019). Polyphenolic flavonoid (Myricetin) upregulated proteasomal degradation mechanisms: eliminates neurodegenerative proteins aggregation. J. Cell Physiol. 234, 20900–20914. 10.1002/jcp.28695 31004355

[B109] Jundt F. Probsting K. S. Anagnostopoulos I. Muehlinghaus G. Chatterjee M. Mathas S.. (2004). Jagged1-induced Notch signaling drives proliferation of multiple myeloma cells. Blood 103, 3511–3515. 10.1182/blood-2003-07-2254 14726396

[B110] Kabashi E. Agar J. N. Taylor D. M. Minotti S. Durham H. D. (2004). Focal dysfunction of the proteasome: a pathogenic factor in a mouse model of amyotrophic lateral sclerosis. J. Neurochem. 89, 1325–1335. 10.1111/j.1471-4159.2004.02453.x 15189335

[B111] Kabashi E. Valdmanis P. N. Dion P. Spiegelman D. McConkey B. J. Velde C. V.. (2008). TARDBP mutations in individuals with sporadic and familial amyotrophic lateral sclerosis. Nat. Genet. 40, 572–574. 10.1038/ng.132 18372902

[B112] Kalchman M. A. Graham R. K. Xia G. Koide H. B. Hodgson J. G. Graham K. C.. (1996). Huntingtin is ubiquitinated and interacts with a specific ubiquitin-conjugating enzyme. J. Biol. Chem. 271, 19385–19394. 10.1074/jbc.271.32.19385 8702625

[B113] Kamura T. Hara T. Kotoshiba S. Yada M. Ishida N. Imaki H.. (2003). Degradation of p57Kip2 mediated by SCFSkp2-dependent ubiquitylation. Proc. Natl. Acad. Sci. U.S.A. 100, 10231–10236. 10.1073/pnas.1831009100 12925736 PMC193544

[B114] Kaur R. Mehan S. Singh S. (2018). Understanding multifactorial architecture of Parkinson's disease: pathophysiology to management. Neurol. Sci. 40, 13–23. 10.1007/s10072-018-3585-x 30267336

[B115] Kazi A. Daniel K. G. Smith D. M. Kumar N. B. Dou Q. P. (2003). Inhibition of the proteasome activity, a novel mechanism associated with the tumor cell apoptosis-inducing ability of genistein. Biochem. Pharmacol. 66, 965–976. 10.1016/s1097-2765(03)00173-4 12963483

[B116] Khasnavis S. Pahan K. (2014). Cinnamon treatment upregulates neuroprotective proteins Parkin and DJ-1 and protects dopaminergic neurons in a mouse model of Parkinson's disease. J. Neuroimmune Pharm. 9, 569–581. 10.1007/s11481-014-9552-2 24946862 PMC4167597

[B117] Kim S. H. Shi Y. Hanson K. A. Williams L. M. Sakasai R. Bowler M. J.. (2009). Potentiation of amyotrophic lateral sclerosis (ALS)-associated TDP-43 aggregation by the proteasome-targeting factor, ubiquilin 1. J. Biol. Chem. 284, 8083–8092. 10.1074/jbc.M808064200 19112176 PMC2658102

[B118] Kim S. Y. Herbst A. Tworkowski K. A. Salghetti S. E. Tansey W. P. (2003). Skp2 regulates Myc protein stability and activity. Mol. Cell 11, 1177–1188. 10.1016/S1097-2765(03)00173-4 12769843

[B119] Kitada T. Pisani A. Porter D. R. Yamaguchi H. Tscherter A. Martella G.. (2007). Impaired dopamine release and synaptic plasticity in the striatum of PINK1-deficient mice. Proc. Natl. Acad. Sci. U.S.A. 104, 11441–11446. 10.1073/pnas.0702717104 17563363 PMC1890561

[B120] Kitada T. Tong Y. Gautier C. A. Shen J. (2009). Absence of nigral degeneration in aged parkin/DJ-1/PINK1 triple knockout mice. J. Neurochem. 111, 696–702. 10.1111/j.1471-4159.2009.06350.x 19694908 PMC2952933

[B121] Klein U. Casola S. Cattoretti G. Shen Q. Lia M. Mo T.. (2006). Transcription factor IRF4 controls plasma cell differentiation and class-switch recombination. Nat. Immunol. 7, 773–782. 10.1038/ni1357 16767092

[B122] Koh S.-H. Lee S. M. Kim H. Y. Lee K.-Y. Lee Y. J. Kim H.-T.. (2006). The effect of epigallocatechin gallate on suppressing disease progression of ALS model mice. Neurosci. Lett. 395, 103–107. 10.1016/j.neulet.2005.10.056 16356650

[B123] Krönke J. Udeshi N. D. Narla A. Grauman P. Hurst S. N. McConkey M.. (2013). Lenalidomide causes selective degradation of IKZF1 and IKZF3 in multiple myeloma cells. Science 343, 301–305. 10.1126/science.1244851 24292625 PMC4077049

[B124] Kumar P. Pradhan K. Karunya R. Ambasta R. K. Querfurth H. W. (2012). Cross-functional E3 ligases Parkin and C-terminus Hsp70-interacting protein in neurodegenerative disorders. J. Neurochem. 120, 350–370. 10.1111/j.1471-4159.2011.07588.x 22098618

[B125] Kumar V. (2006). Potential medicinal plants for CNS disorders: an overview. Phytother. Res. 20, 1023–1035. 10.1002/ptr.1970 16909441

[B126] Kwak M.-K. Wakabayashi N. Greenlaw J. L. Yamamoto M. Kensler T. W. (2003). Antioxidants enhance mammalian proteasome expression through the Keap1-Nrf2 signaling pathway. Mol. Cell Biol. 23, 8786–8794. 10.1128/mcb.23.23.8786-8794.2003 14612418 PMC262680

[B127] Kwiatkowski T. J. Bosco D. Leclerc A. Tamrazian E. Vanderburg C. Russ C.. (2009). Mutations in the FUS/TLS gene on chromosome 16 cause familial amyotrophic lateral sclerosis. Science 323, 1205–1208. 10.1126/science.1166066 19251627

[B128] Lee B.-H. Lee M. J. Park S. Oh D.-C. Elsasser S. Chen P.-C.. (2010). Enhancement of proteasome activity by a small-molecule inhibitor of USP14. Nature 467, 179–184. 10.1038/nature09299 20829789 PMC2939003

[B129] Lee K. M. Jo S. Kim H. Lee J. Park C.-S. (2011). Functional modulation of AMP-activated protein kinase by cereblon. Biochim. Biophys. Acta Mol. Cell Res. 1813, 448–455. 10.1016/j.bbamcr.2011.01.005 21232561

[B130] Lee M. J. Lee J. H. Rubinsztein D. C. (2013). Tau degradation: the ubiquitin–proteasome system versus the autophagy-lysosome system. Prog. Neurobiol. 105, 49–59. 10.1016/j.pneurobio.2013.03.001 23528736

[B131] Leestemaker Y. Ovaa H. (2017). Tools to investigate the ubiquitin proteasome system. Drug Discov. Today Technol. 26, 25–31. 10.1016/j.ddtec.2017.11.006 29249239

[B132] Leigh P. Whitwell H. Garofalo O. Buller J. Swash M. Martin J.. (1991). Ubiquitin-immunoreactive intraneuronal inclusions in amyotrophic lateral sclerosis: morphology, distribution, and specificity. Brain 114, 775–788. 10.1093/brain/114.2.775 1646064

[B133] Li M. Brooks C. L. Kon N. Gu W. (2004). A dynamic role of HAUSP in the p53-Mdm2 pathway. Mol. Cell 13, 879–886. 10.1016/s1097-2765(04)00157-1 15053880

[B134] Li T. Feng Y. Yang R. Wu L. Li R. Huang L.. (2018). Salidroside promotes the pathological α-synuclein clearance through ubiquitin-proteasome system in SH-SY5Y cells. Front. Pharmacol. 9:377. 10.3389/fphar.2018.00377 29725300 PMC5917065

[B135] Li X. Zhou J. Chen H. Wang F. Mei Q. Sun H. (2017). The association between the UBQLN1 polymorphism and Alzheimer's disease risk: a systematic review. Cell Mol. Biol. 63, 94–96. 10.14715/cmb/2017.63.5.17 28719358

[B136] Lim K.-L. Dawson V. L. Dawson T. M. (2006). Parkin-mediated lysine 63-linked polyubiquitination: a link to protein inclusions formation in Parkinson's and other conformational diseases? Neurobiol. Aging 27, 524–529. 10.1016/j.neurobiolaging.2005.07.023 16213628

[B137] Lin C. W. Wu M. J. Liu I. Y. Su J. D. Yen J. H. (2010). Neurotrophic and cytoprotective action of luteolin in PC12 cells through ERK dependent induction of Nrf2-driven HO-1 expression. J. Agric. Food Chem. 58, 4477–4486 10.1021/jf904061x 20302373

[B138] Liu J. Shaik S. Dai X. Wu Q. Zhou X. Wang Z.. (2015). Targeting the ubiquitin pathway for cancer treatment. Biochim. Biophys. Acta Rev. Cancer 1855, 50–60. 10.1016/j.bbcan.2014.11.005 25481052 PMC4312704

[B139] Liu W. Vives-Bauza C. Yamamoto A. Tan Y. Li Y. Magrané J.. (2009). PINK1 defect causes mitochondrial dysfunction, proteasomal deficit and α-synuclein aggregation in cell culture models of Parkinson's disease. PLoS ONE 4:e4597. 10.1371/journal.pone.0004597 19242547 PMC2644779

[B140] Liu Y. Hettinger C. L. Zhang D. Rezvani K. Wang X. Wang H. (2014). The proteasome function reporter GFPu accumulates in young brains of the APPswe/PS1dE9 Alzheimer's disease mouse model. Cell Prog. Neurobiol. 34, 315–322. 10.1007/s10571-013-0022-9 24363091 PMC3954921

[B141] Lobo V. Patil A. Phatak A. Chandra N. (2010). Free radicals, antioxidants and functional foods: impact on human health. Pharmacogn. Rev. 4, 118–126. 10.4103/0973-7847.70902 22228951 PMC3249911

[B142] Logroscino G. Traynor B. J. Hardiman O. Chiò A. Mitchell D. Swingler R. J.. (2010). Incidence of amyotrophic lateral sclerosis in Europe. J. Neurol. Neurosurg. Psychiatry 81:385–390. 10.1136/jnnp.2009.183525 19710046 PMC2850819

[B143] Lorenc-Koci E. Lenda T. Antkiewicz-Michaluk L. Wardas J. Domin H. Smiałowska M.. (2011). Different effects of intranigral and intrastriatal administration of the proteasome inhibitor lactacystin on typical neurochemical and histological markers of Parkinson's disease in rats. Neurochem. Int. 58, 839–849. 10.1016/j.neuint.2011.03.013 21419185

[B144] Lunati A. Lesage S. Brice A. (2018). The genetic landscape of Parkinson's disease. Rev. Neurol. 174, 628–643. 10.1016/j.neurol.2018.08.004 30245141

[B145] Ma M. H. Yang H. H. Parker K. Manyak S. Friedman J. M. Altamirano C.. (2003). The proteasome inhibitor PS-341 markedly enhances sensitivity of multiple myeloma tumor cells to chemotherapeutic agents. Clin. Cancer Res. 9, 1136–1144. 12631619

[B146] Manavalan A. Mishra M. Feng L. Sze S. K. Akatsu H. Heese K. (2013). Brain site-specific proteome changes in aging-related dementia. Exp. Mol. Med. 45:e39. 10.1038/emm.2013.76 24008896 PMC3789264

[B147] Mandel S. A. Amit T. Weinreb O. Youdim M. B. (2011). Understanding the broad-spectrum neuroprotective action profile of green tea polyphenols in aging and neurodegenerative diseases. J. Alzheimers Dis. 25, 187–208. 10.3233/JAD-2011-101803 21368374

[B148] Marambaud P. Zhao H. Davies P. (2005). Resveratrol promotes clearance of Alzheimer's disease amyloid-β peptides J. Biol. Chem. 280, 37377–37382. 10.1074/jbc.M508246200 16162502

[B149] Martin D. D. Ladha S. Ehrnhoefer D. E. Hayden M. R. (2015). Autophagy in Huntington disease and huntingtin in autophagy. Trends Neurosci. 38, 26–35. 10.1016/j.tins.2014.09.003 25282404

[B150] Martin L. Latypova X. Terro F. (2011). Post-translational modifications of tau protein: implications for Alzheimer's disease. Neurochem. Int. 58, 458–471. 10.1016/j.neuint.2010.12.023 21215781

[B151] Martínez-Huélamo M. Rodríguez-Morató J. Boronat A. de la Torre R. (2017). Modulation of Nrf2 by olive oil and wine polyphenols and neuroprotection. Antioxidants 6:73. 10.3390/antiox6040073 28954417 PMC5745483

[B152] Maruyama H. Morino H. Ito H. Izumi Y. Kato H. Watanabe Y.. (2010). Mutations of optineurin in amyotrophic lateral sclerosis. Nature 465:223. 10.1038/nature08971 20428114

[B153] Massey L. K. Mah A. L. Ford D. L. Miller J. Liang J. Doong H.. (2004). Overexpression of ubiquilin decreases ubiquitination and degradation of presenilin proteins. J. Alzheimers Dis. 6, 79–92. 10.3233/jad-2004-6109 15004330

[B154] Minati L. Edginton T. Grazia Bruzzone M. Giaccone G. (2009). Reviews: current concepts in Alzheimer's disease: a multidisciplinary review. Am. J. Alzheimers Dis. Other Dement. 24, 95–121. 10.1177/1533317508328602 PMC10846154 19116299

[B155] Mines M. A. Goodwin J. S. Limbird L. E. Cui F.-F. Fan G.-H. (2009). Deubiquitination of CXCR4 by USP14 is critical for both CXCL12-induced CXCR4 degradation and chemotaxis but not ERK activation. J. Biol. Chem. 284, 5742–5752. 10.1074/jbc.M808507200 19106094 PMC2645827

[B156] Mitsiades N. Mitsiades C. S. Poulaki V. Chauhan D. Richardson P. G. Hideshima T.. (2002). Biologic sequelae of nuclear factor–κB blockade in multiple myeloma: therapeutic applications. Blood 99, 4079–4086. 10.1182/blood.v99.11.4079 12010810

[B157] Mittrücker H.-W. Matsuyama T. Grossman A. Kündig T. M. Potter J. Shahinian A.. (1997). Requirement for the transcription factor LSIRF/IRF4 for mature B and T lymphocyte function. Science 275, 540–543. 10.1126/science.275.5299.540 8999800

[B158] Moosavi F. Hosseini R. Saso L. Firuzi O. (2016). Modulation of neurotrophic signaling pathways by polyphenols. Drug Des. Dev. Ther. 10, 23–42. 10.2147/DDDT.S96936 26730179 PMC4694682

[B159] Morawe T. Hiebel C. Kern A. Behl C. (2012). Protein homeostasis, aging and Alzheimer's disease. Mol. Neurobiol. 46, 41–54. 10.1007/s12035-012-8246-0 22361852 PMC3443483

[B160] Morgan G. J. Walker B. A. Davies F. E. (2012). The genetic architecture of multiple myeloma. Nat. Rev. Cancer 12:335. 10.1038/nrc3257 22495321

[B161] Mori F. Nishie M. Piao Y. S. Kito K. Kamitani T. Takahashi H.. (2005). Accumulation of NEDD8 in neuronal and glial inclusions of neurodegenerative disorders. Neuropathol. Appl. Neurobiol. 31, 53–61. 10.1111/j.1365-2990.2004.00603.x 15634231

[B162] Moussa C. Hebron M. Huang X. Ahn J. Rissman R. A. Aisen P. S.. (2017). Resveratrol regulates neuro-inflammation and induces adaptive immunity in Alzheimer's disease. J. Neuroinflamm. 14:1. 10.1186/s12974-016-0779-0 28086917 PMC5234138

[B163] Mullally J. Fitzpatrick F. (2002). Pharmacophore model for novel inhibitors of ubiquitin isopeptidases that induce p53-independent cell death. Mol. Pharmacol. 62, 351–358. 10.1124/mol.62.2.351 12130688

[B164] Murakami A. (2013). Modulation of protein quality control systems by food phytochemicals. J. Clin. Biochem. Nutr. 52, 215–227. 10.3164/jcbn.12-126 23704811 PMC3652296

[B165] Nabavi S. F. Atanasov A. G. Khan H. Barreca D. Trombetta D. Testai L.. (2018a). Targeting ubiquitin-proteasome pathway by natural, in particular polyphenols, anticancer agents: lessons learned from clinical trials. Cancer Lett. 434, 101–113. 10.1016/j.canlet.2018.07.018 30030139

[B166] Nabavi S. F. Daglia M. D'Antona G. Sobarzo-Sanchez E. Talas Z. S. Nabavi S. M. (2015). Natural compounds used as therapies targeting to amyotrophic lateral sclerosis. Curr. Pharm. Biotechnol. 16, 211–218. 10.2174/1389201016666150118132224 25601606

[B167] Nabavi S. F. Sureda A. Dehpour A. R. Shirooie S. Silva A. S. Devi K. P.. (2018b). Regulation of autophagy by polyphenols: paving the road for treatment of neurodegeneration. Biotechnol. Adv. 36, 1768–1778. 10.1016/j.biotechadv.2017.12.001 29221764

[B168] Nam S. Smith D. M. Dou Q. P. (2001). Ester bond-containing tea polyphenols potently inhibit proteasome activity *in vitro* and *in vivo*. J. Biol. Chem. 276, 13322–13330. 10.1074/jbc.M004209200 11278274

[B169] N'Diaye E.-N. Debnath J. Brown E. J. (2009). Ubiquilins accelerate autophagosome maturation and promote cell survival during nutrient starvation. Autophagy 5, 573–575. 10.4161/auto.5.4.8312 19398896

[B170] Neumann M. Sampathu D. M. Kwong L. K. Truax A. C. Micsenyi M. C. Chou T. T.. (2006). Ubiquitinated TDP-43 in frontotemporal lobar degeneration and amyotrophic lateral sclerosis. Science 314, 130–133. 10.1126/science.1134108 17023659

[B171] Nicholson B. Marblestone J. G. Butt T. R. Mattern M. R. (2007). Deubiquitinating enzymes as novel anticancer targets. Future Oncol. 3, 191–199. 10.2217/14796694.3.2.191 17381419 PMC2291548

[B172] Nural H. He P. Beach T. Sue L. Xia W. Shen Y. (2009). Dissembled DJ-1 high molecular weight complex in cortex mitochondria from Parkinson's disease patients. Mol. Neurodegener. 4:23. 10.1186/1750-1326-4-23 19497122 PMC2704189

[B173] Obeng E. A. Carlson L. M. Gutman D. M. Harrington W. J. Lee K. P. Boise L. H. (2006). Proteasome inhibitors induce a terminal unfolded protein response in multiple myeloma cells. Blood 107, 4907–4916. 10.1182/blood-2005-08-3531 16507771 PMC1895817

[B174] Oddo S. (2008). The ubiquitin-proteasome system in Alzheimer's disease. J. Cell Mol. Med. 12, 363–373. 10.1111/j.1582-4934.2008.00276.x 18266959 PMC3822529

[B175] Oddo S. Caccamo A. Tran L. Lambert M. P. Glabe C. G. Klein W. L.. (2006). Temporal profile of amyloid-β (Aβ) oligomerization in an *in vivo* model of Alzheimer disease a link between Aβ and tau pathology. J. Biol. Chem. 281, 1599–1604. 10.1074/jbc.M507892200 16282321

[B176] Okamoto K. Hirai S. Yamazaki T. Sun X. Nakazato Y. (1991). New ubiquitin-positive intraneuronal inclusions in the extra-motor cortices in patients with amyotrophic lateral sclerosis. Neurosci. Lett. 129, 233–236. 10.1016/0304-3940(91)90469-a 1660578

[B177] Olzmann J. A. Li L. Chin L. S. (2008). Aggresome formation and neurodegenerative diseases: therapeutic implications. Curr. Med. Chem. 15, 47–60. 10.2174/092986708783330692 18220762 PMC4403008

[B178] Orhan I. Daglia M. Nabavi S. Loizzo M. Sobarzo-Sanchez E. Nabavi S. (2015). Flavonoids and dementia: an update. Curr. Med. Chem. 22, 1004–1015. 10.2174/0929867322666141212122352 25515512

[B179] Orlowski M. Wilk S. (2000). Catalytic activities of the 20 S proteasome, a multicatalytic proteinase complex. Arch. Biochem. Biophys. 383, 1–16. 10.1006/abbi.2000.2036 11097171

[B180] Pandareesh M. Mythri R. Bharath M. S. (2015). Bioavailability of dietary polyphenols: factors contributing to their clinical application in CNS diseases. Neurochem. Int. 89, 198–208. 10.1016/j.neuint.2015.07.003 26163045

[B181] Peña-Altamira E. Petralla S. Massenzio F. Virgili M. Bolognesi M. L. Monti B. (2017). Nutritional and pharmacological strategies to regulate microglial polarization in cognitive aging and Alzheimer's disease. Front. Aging Neurosci. 9:175. 10.3389/fnagi.2017.00175 28638339 PMC5461295

[B182] Poon W. W. Carlos A. J. Aguilar B. L. Berchtold N. C. Kawano C. K. Zograbyan V.. (2013). β-Amyloid (Aβ) oligomers impair brain-derived neurotrophic factor retrograde trafficking by down-regulating ubiquitin C-terminal hydrolase, UCH-L1. J. Biol. Chem. 288, 16937–16948. 10.1074/jbc.M113.463711 23599427 PMC3675626

[B183] Popovic D. Vucic D. Dikic I. (2014). Ubiquitination in disease pathogenesis and treatment. Nat. Med. 20:1242. 10.1038/nm.3739 25375928

[B184] Querfurth H. W. LaFerla F. M. (2010). Alzheimer's disease. N. Engl. J. Med. 362, 329–344. 10.1056/NEJMra0909142 20107219

[B185] Rahman I. Chung S. (2010). Dietary polyphenols, deacetylases and chromatin remodeling in resveratrol and other polyphenols on the most common brain age-related diseases. Curr. Med. Chem. 24, 4245–4266. 10.1159/000314513 28738770

[B186] Rao G. Croft B. Teng C. Awasthi V. (2015). Ubiquitin-proteasome system in neurodegenerative disorders. J. Drug Metab. Toxicol. 6:187. 10.4172/2157-7609.1000187 30761219 PMC6370320

[B187] Renton A. E. Majounie E. Waite A. Simón-Sánchez J. Rollinson S. Gibbs J. R.. (2011). A hexanucleotide repeat expansion in C9ORF72 is the cause of chromosome 9p21-linked ALS-FTD. Neuron 72, 257–268. 10.1016/j.neuron.2011.09.010 21944779 PMC3200438

[B188] Reyes-Turcu F. E. Ventii K. H. Wilkinson K. D. (2009). Regulation and cellular roles of ubiquitin-specific deubiquitinating enzymes. Ann. Rev. Biochem. 78, 363–397. 10.1146/annurev.biochem.78.082307.091526 19489724 PMC2734102

[B189] Richardson P. G. Hideshima T. Anderson K. C. (2003). Bortezomib (PS-341): a novel, first-in-class proteasome inhibitor for the treatment of multiple myeloma and other cancers. Cancer Control 10, 361–369. 10.1177/107327480301000502 14581890

[B190] Ringman J. M. Frautschy S. A. Teng E. Begum A. N. Bardens J. Beigi M.. (2012). Oral curcumin for Alzheimer's disease: tolerability and efficacy in a 24-week randomized, double blind, placebo-controlled study. Alzheimers Res. Ther. 4:43. 10.1186/alzrt146 23107780 PMC3580400

[B191] Rivett A. J. Hearn A. R. (2004). Proteasome function in antigen presentation: immunoproteasome complexes, peptide production, and interactions with viral proteins. Curr. Protein Pept. Sci. 5, 153–161. 10.2174/1389203043379774 15180520

[B192] Rock K. L. Gramm C. Rothstein L. Clark K. Stein R. Dick L.. (1994). Inhibitors of the proteasome block the degradation of most cell proteins and the generation of peptides presented on MHC class I molecules. Cell 78, 761–771. 10.1016/s0092-8674(94)90462-6 8087844

[B193] Rock K. L. York I. A. Saric T. Goldberg A. L. (2002). Protein degradation and the generation of MHC class I-presented peptides Adv. Immunol. 80, 1–70. 10.1016/S0065-2776(02)80012-8 12078479

[B194] Rosen D. R. Siddique T. Patterson D. Figlewicz D. A. Sapp P. Hentati A.. (1993). Mutations in Cu/Zn superoxide dismutase gene are associated with familial amyotrophic lateral sclerosis. Nature 362:59. 10.1038/362059a0 8446170

[B195] Russo S. M. Tepper J. E. Baldwin A. S. Jr. Liu R. Adams J. Elliott P.. (2001). Enhancement of radiosensitivity by proteasome inhibition: implications for a role of NF-κB. Int. J. Radiat. Oncol. Biol. Phys. 50, 183–193. 10.1016/s0360-3016(01)01446-8 11316563

[B196] Safren N. Chang L. Dziki K. M. Monteiro M. J. (2015). Signature changes in ubiquilin expression in the R6/2 mouse model of Huntington's disease. Brain Res. 1597, 37–46. 10.1016/j.brainres.2014.12.008 25511991 PMC4340744

[B197] Salminen A. Kaarniranta K. Haapasalo A. Hiltunen M. Soininen H. Alafuzoff I. (2012). Emerging role of p62/sequestosome-1 in the pathogenesis of Alzheimer's disease. Prog. Neurobiol. 96, 87–95. 10.1016/j.pneurobio.2011.11.005 22138392

[B198] Sanadgol N. Zahedani S. S. Sharifzadeh M. Khalseh R. Barbari G. R. Abdollahi M. (2017). Recent updates in imperative natural compounds for healthy brain and nerve function: a systematic review of implications for multiple sclerosis. Curr. Drug Targets 18, 1499–1517. 10.2174/1389450118666161108124414 27829351

[B199] Sang Y. Yan F. Ren X. (2015). The role and mechanism of CRL4 E3 ubiquitin ligase in cancer and its potential therapy implications. Oncotarget 6, 42590–42602. 10.18632/oncotarget.6052 26460955 PMC4767455

[B200] Sarubbo F. Moranta D. Asensio V. J. Miralles A. Esteban S. (2017). Effects of resveratrol and other polyphenols on the most common brain age-related diseases. Curr. Med. Chem. 24, 4245–4266. 10.2174/0929867324666170724102743 28738770

[B201] Saurin A. J. Borden K. L. Boddy M. N. Freemont P. S. (1996). Does this have a familiar RING? Trends Biochem. Sci. 21, 208–214. 10.1016/S0968-0004(96)80017-X 8744354

[B202] Scherer D. C. Brockman J. A. Bendall H. H. Zhang G. M. Ballard D. W. Oltz E. M. (1996). Corepression of RelA and c-Rel inhibits immunoglobulin κ gene transcription and rearrangement in precursor B lymphocytes. Immunity 5, 563–574. 10.1016/s1074-7613(00)80271-x 8986716

[B203] Sciammas R. Shaffer A. Schatz J. H. Zhao H. Staudt L. M. Singh H. (2006). Graded expression of interferon regulatory factor-4 coordinates isotype switching with plasma cell differentiation. Immunity 25, 225–236. 10.1016/j.immuni.2006.07.009 16919487

[B204] Selkoe D. J. (2002). Alzheimer's disease is a synaptic failure. Science 298, 789–791. 10.1126/science.1074069 12399581

[B205] Seo H. Isacson O. (2010). The hAPP-YAC transgenic model has elevated UPS activity in the frontal cortex similar to Alzheimer's disease and Down's syndrome. J. Neurochem. 114, 1819–1826. 10.1111/j.1471-4159.2010.06902.x 20698932

[B206] Setsuie R. Wada K. (2007). The functions of UCH-L1 and its relation to neurodegenerative diseases. Neurochem. Int. 51, 105–111. 10.1016/j.neuint.2007.05.007 17586089

[B207] Shaffer A. L. Emre N. C. Lamy L. Ngo V. N. Wright G. Xiao W.. (2008). IRF4 addiction in multiple myeloma. Nature 454, 226–231. 10.1038/nature07064 18568025 PMC2542904

[B208] Shinbo Y. Niki T. Taira T. Ooe H. Takahashi-Niki K. Maita C.. (2006). Proper SUMO-1 conjugation is essential to DJ-1 to exert its full activities. Cell Death Differ. 13, 96–108. 10.1038/sj.cdd.4401704 15976810

[B209] Shinji S. Naito Z. Ishiwata S. Ishiwata T. Tanaka N. Furukawa K.. (2006). Ubiquitin-specific protease 14 expression in colorectal cancer is associated with liver and lymph node metastases. Oncol. Rep. 15, 539–543. 10.3892/or.15.3.539 16465409

[B210] Si X. Wang Y. Wong J. Zhang J. McManus B. M. Luo H. (2007). Dysregulation of the ubiquitin-proteasome system by curcumin suppresses coxsackievirus B3 replication. J. Virol. 81, 3142–3150. 10.1128/JVI.02028-06 17229707 PMC1866032

[B211] Smith D. M. Wang Z. Kazi A. Li L.-H. Chan T.-H. Dou Q. P. (2002). Synthetic analogs of green tea polyphenols as proteasome inhibitors. Mol. Med. 8:382. 10.1007/BF03402019 12393936 PMC2040000

[B212] Snyder H. Mensah K. Theisler C. Lee J. M. Matouschek A. Wolozin B. (2003). Aggregated and monomeric alpha-synuclein bind to the S6'proteasomal protein and inhibit proteasomal function. J. Biol. Chem. 278, 11753–11759. 10.1074/jbc.M208641200 12551928

[B213] Song M. Song S. Kim S. Nakayama K. Nakayama K. Lim D.-S. (2008). Skp2 regulates the antiproliferative function of the tumor suppressor RASSF1A via ubiquitin-mediated degradation at the G 1–S transition. Oncogene 27:3176 10.1038/sj.onc.1210971 18071316

[B214] Song Y. J. C. Halliday G. M. Holton J. L. Lashley T. O'sullivan S. S. McCann H.. (2009). Degeneration in different parkinsonian syndromes relates to astrocyte type and astrocyte protein expression. J. Neuropathol. Exp. Neurol. 68, 1073–1083. 10.1097/NEN.0b013e3181b66f1b 19918119

[B215] Srivastava P. Dhuriya Y. K. Kumar V. Srivastava A. Gupta R. Shukla R. K.. (2018). PI3K/Akt/GSK3β induced CREB activation ameliorates arsenic mediated alterations in NMDA receptors and associated signaling in rat hippocampus: neuroprotective role of curcumin. Neurotoxicology 67, 190–205. 10.1016/j.neuro.2018.04.018 29723552

[B216] Stieren E. S. El Ayadi A. Xiao Y. Siller E. Landsverk M. L. Oberhauser A. F.. (2011). Ubiquilin-1 is a molecular chaperone for the amyloid precursor protein. J. Biol. Chem. 286, 35689–35698. 10.1074/jbc.M111.243147 21852239 PMC3195644

[B217] Stolz A. Dikic I. (2018). Heterotypic ubiquitin chains: seeing is believing. Trends Cell Biol. 28, 1–3. 10.1016/j.tcb.2017.11.005 29191367

[B218] Strathearn K. E. Yousef G. G. Grace M. H. Roy S. L. Tambe M. A. Ferruzzi M. G. (2014). Neuroprotective effects of anthocyanin-and proanthocyanidin-rich extracts in cellular models of Parkinson? s disease. Brain Res. 1555, 60–77. 10.1016/j.brainres.2014.01.047 24502982 PMC4024464

[B219] Sundaram R. S. Gowtham L. (2012). Microglia and regulation of inflammation-mediated neurodegeneration: prevention and treatment by phytochemicals and metabolic nutrients. Int. J. Green Pharm. 6, 81–92. 10.4103/0973-8258.102807

[B220] Sunwoo J. B. Chen Z. Dong G. Yeh N. Bancroft C. C. Sausville E. (2001). Novel proteasome inhibitor PS-341 inhibits activation of nuclear factor-κB, cell survival, tumor growth, and angiogenesis in squamous cell carcinoma. Clin. Cancer Res. 7, 1419–1428. 11350913

[B221] Tai H.-C. Serrano-Pozo A. Hashimoto T. Frosch M. P. Spires-Jones T. L. Hyman B. T. (2012). The synaptic accumulation of hyperphosphorylated tau oligomers in Alzheimer disease is associated with dysfunction of the ubiquitin-proteasome system. Am. J. Pathol. 181, 1426–1435. 10.1016/j.ajpath.2012.06.033 22867711 PMC3463637

[B222] Tambe M. A. (2015). Neuroprotective effects of polyphenols in cellular models of Parkinson's disease. Brain Res. 1555, 60–77. 10.1016/j.brainres.2015.01.047 PMC4024464 24502982

[B223] Tan S. H. Karri V. Tay N. W. R. Chang K. H. Ah H. Y. Ng P. Q.. (2019). Emerging pathways to neurodegeneration: dissecting the critical molecular mechanisms in Alzheimer's disease, Parkinson's disease. Biomed. Pharmacother. 111, 765–777. 10.1016/j.biopha.2018.12.101 30612001

[B224] Tanaka K. Suzuki T. Chiba T. (1998). The ligation systems for ubiquitin and ubiquitin-like proteins. Mol. Cells 8, 503–512. 9856335

[B225] Tanaka Y. Engelender S. Igarashi S. Rao R. K. Wanner T. Tanzi R. E.. (2001). Inducible expression of mutant α-synuclein decreases proteasome activity and increases sensitivity to mitochondria-dependent apoptosis. Hum. Mol. Genet. 10, 919–926. 10.1093/hmg/10.9.919 11309365

[B226] Tashiro Y. Urushitani M. Inoue H. Koike M. Uchiyama Y. Komatsu M. (2012). Motor neuron-specific disruption of proteasomes, but not autophagy, replicates amyotrophic lateral sclerosis. J. Biol. Chem. 112:417600 10.1074/jbc.M112.417600 PMC3522293 23095749

[B227] Tian Z. D'Arcy P. Wang X. Ray A. Tai Y.-T. Hu Y.. (2013). A novel small molecule inhibitor of deubiquitylating enzyme USP14 and UCHL5 induces apoptosis in multiple myeloma and overcomes bortezomib resistance. Blood 123, 706–716. 10.1182/blood-2013-05-500033 24319254 PMC3907756

[B228] Todi S. V. Winborn B. J. Scaglione K. M. Blount J. R. Travis S. M. Paulson H. L. (2009). Ubiquitination directly enhances activity of the deubiquitinating enzyme ataxin-3. EMBO. J. 28, 372–382. 10.1038/emboj.2008.289 19153604 PMC2646149

[B229] Tosukhowong P. Boonla C. Dissayabutra T. Kaewwilai L. Muensri S. Chotipanich C.. (2016). Biochemical and clinical effects of Whey protein supplementation in Parkinson's disease: a pilot study. J. Neurol. Sci. 367, 162–170. 10.1016/j.jns.2016.05.056 27423583

[B230] Tseng B. P. Green K. N. Chan J. L. Blurton-Jones M. LaFerla F. M. (2008). Aβ inhibits the proteasome and enhances amyloid and tau accumulation. Neurobiol. Aging 29, 1607–1618. 10.1016/j.neurobiolaging.2007.04.014 17544172 PMC2664168

[B231] Tsvetkov L. M. Yeh K.-H. Lee S.-J. Sun H. Zhang H. (1999). p27Kip1 ubiquitination and degradation is regulated by the SCFSkp2 complex through phosphorylated Thr187 in p27. Curr. Biol. 9, 661–S662. 10.1016/S0960-9822(99)80290-5 10375532

[B232] Turner R. S. Thomas R. G. Craft S. van Dyck C. H. Mintzer J. Reynolds B. A.. (2015). A randomized, double-blind, placebo-controlled trial of resveratrol for Alzheimer disease. Neurology 85, 1383–1391. 10.1212/WNL.0000000000002035 26362286 PMC4626244

[B233] Urushitani M. Kurisu J. Tsukita K. Takahashi R. (2002). Proteasomal inhibition by misfolded mutant superoxide dismutase 1 induces selective motor neuron death in familial amyotrophic lateral sclerosis. J. Neurochem. 83, 1030–1042. 10.1046/j.1471-4159.2002.01211.x 12437574

[B234] Valenti D. de Bari L. de Rasmo D. Signorile A. Henrion-Caude A. Contestabile A.. (2016). The polyphenols resveratrol and epigallocatechin-3-gallate restore the severe impairment of mitochondria in hippocampal progenitor cells from a down syndrome mouse model. Biochim. Biophys. Acta 1862, 1093–1104. 10.1016/j.bbadis.2016.03.003 26964795

[B235] Van Deerlin V. M. Leverenz J. B. Bekris L. M. Bird T. D. Yuan W. Elman L. B.. (2008). TARDBP mutations in amyotrophic lateral sclerosis with TDP-43 neuropathology: a genetic and histopathological analysis. Lancet Neurol. 7, 409–416. 10.1016/S1474-4422(08)70071-1 18396105 PMC3546119

[B236] van Horssen J. Drexhage J. A. Flor T. Gerritsen W. van der Valk P. de Vries H. E. (2010). Nrf2 and DJ1 are consistently upregulated in inflammatory multiple sclerosis lesions. Free Radic. Biol. Med. 49, 1283–1289. 10.1016/j.freeradbiomed.2010.07.013 20673799

[B237] Vance C. Rogelj B. Hortobágyi T. De Vos K. J. Nishimura A. L. Sreedharan J.. (2009). Mutations in FUS, an RNA processing protein, cause familial amyotrophic lateral sclerosis type 6. Science 323, 1208–1211. 10.1126/science.1165942 19251628 PMC4516382

[B238] Von Der Lehr N. Johansson S. Wu S. Bahram F. Castell A. Cetinkaya C.. (2003). The F-box protein Skp2 participates in c-Myc proteosomal degradation and acts as a cofactor for c-Myc-regulated transcription. Mol. Cell 11, 1189–1200. 10.1016/s1097-2765(03)00193-x 12769844

[B239] Wagner S. Carpentier I. Rogov V. Kreike M. Ikeda F. Löhr F.. (2008). Ubiquitin binding mediates the NF-κB inhibitory potential of ABIN proteins. Oncogene 27:3739. 10.1038/sj.onc.1211042 18212736

[B240] Wang C. Shou Y. Pan J. Du Y. Liu C. Wang H. (2018). The relationship between cholesterol level and Alzheimer's disease-associated APP proteolysis/Abeta metabolism. Nutr. Neurosci. 22, 453–463. 10.1080/1028415X.2017.1416942 29325505

[B241] Wang H.-Y. Wang I.-F. Bose J. Shen C.-K. J. (2004). Structural diversity and functional implications of the eukaryotic TDP gene family. Genomics 83, 130–139. 10.1016/s0888-7543(03)00214-3 14667816

[B242] Wang R. Ying Z. Zhao J. Zhang Y. Wang R. Lu H. (2012). Lys203 and Lys382 are essential for the proteasomal degradation of BACE1. Curr. Alzheimer Res. 9, 606–615. 10.2174/156720512800618026 22299711

[B243] Wang X. Mazurkiewicz M. Hillert E.-K. Olofsson M. H. Pierrou S. Hillertz P. (2016). The proteasome deubiquitinase inhibitor VLX1570 shows selectivity for ubiquitin-specific protease-14 and induces apoptosis of multiple myeloma cells. Sci. Rep. 6:26979 10.1038/srep26979 27264969 PMC4893612

[B244] Wang Y. Garg S. Mandelkow E.-M. Mandelkow E. (2010). Proteolytic processing of tau. Biochem.ss Soc. Trans. 38, 955–961. 10.1042/BST0380955 20658984

[B245] Waring S. C. Rosenberg R. N. (2008). Genome-wide association studies in Alzheimer disease. Arch. Neurol. 65, 329–334. 10.1001/archneur.65.3.329 18332245

[B246] Wickner S. Maurizi M. R. Gottesman S. (1999). Posttranslational quality control: folding, refolding, and degrading proteins. Science 286, 1888–1893. 10.1126/science.286.5446.1888 10583944

[B247] Wicks S. J. Haros K. Maillard M. Song L. Cohen R. E. ten Dijke P.. (2005). The deubiquitinating enzyme UCH37 interacts with Smads and regulates TGF-β signalling. Oncogene 24:8080. 10.1038/sj.onc.1208944 16027725

[B248] William C. S. Liou H.-C. Tuomanen E. I. Baltimore D. (1995). Targeted disruption of the p50 subunit of NF-κB leads to multifocal defects in immune responses. Cell 80, 321–330. 10.1016/0092-8674(95)90415-8 7834752

[B249] Williams K. L. Topp S. Yang S. Smith B. Fifita J. A. Warraich S. T.. (2016). CCNF mutations in amyotrophic lateral sclerosis and frontotemporal dementia. Nat. Commun. 7:11253. 10.1038/ncomms11253 27080313 PMC4835537

[B250] Winklhofer K. F. Haass C. (2010). Mitochondrial dysfunction in Parkinson's disease. Biochim. Biophys. Acta 1802, 29–44. 10.1016/j.bbadis.2009.08.013 19733240

[B251] Wu M. Lee H. Bellas R. E. Schauer S. L. Arsura M. Katz D.. (1996). Inhibition of NF-kappaB/Rel induces apoptosis of murine B cells. EMBO J. 15, 4682–4690. 10.1002/j.1460-2075.1996.tb00845.x 8887559 PMC452200

[B252] Xie H. Wu J. (2016). Silica nanoparticles induce alpha-synuclein induction and aggregation in PC12-cells. Chem. Biol. Interact. 258, 197–204. 10.1016/j.cbi.2016.09.006 27613482

[B253] Xiong H. Wang D. Chen L. Choo Y. S. Ma H. Tang C.. (2009). Parkin, PINK1, and DJ-1 form a ubiquitin E3 ligase complex promoting unfolded protein degradation. J. Clin. Invest. 119, 650–660. 10.1172/JCI37617 19229105 PMC2648688

[B254] Xue S. Jia J. (2006). Genetic association between ubiquitin carboxy-terminal hydrolase-L1 gene S18Y polymorphism and sporadic Alzheimer's disease in a Chinese Han population. Brain Res. 1087, 28–32. 10.1016/j.brainres.2006.02.121 16626667

[B255] Yassa N. Masoomi F. Rohani Rankouhi S. E. Hadjiakhoondi A. (2009). Chemical composition and antioxidant activity of the extract and essential oil of Rosa damascena from Iran, population of Guilan. Daru 17, 175–180.

[B256] Yin F. Ye F. Tan L. Liu K. Xuan Z. Zhang J.. (2012). Alterations of signaling pathways in muscle tissues of patients with amyotrophic lateral sclerosis. Muscle Nerve 46, 856–860. 10.1002/mus.23411 22996383

[B257] Yu Z.-K. Gervais J. L. Zhang H. (1998). Human CUL-1 associates with the SKP1/SKP2 complex and regulates p21CIP1/WAF1 and cyclin D proteins. Proc. Natl. Acad. Sci. U.S.A. 95, 11324–11329. 10.1073/pnas.95.19.11324 9736735 PMC21641

[B258] Zaffagnini G. Savova A. Danieli A. Romanov J. Tremel S. Ebner M. (2018). Phasing out the bad-How SQSTM1/p62 sequesters ubiquitinated proteins for degradation by autophagy. Autophagy 14, 1280–1282. 10.1080/15548627.2018.1462079 29929426 PMC6103668

[B259] Zeng X. S. Geng W. S. Jia J. J. Chen L. Zhang P. P. (2018). Cellular and molecular basis of neurodegeneration in parkinson disease. Front. Aging Neurosci. 10:109. 10.3389/fnagi.2018.00109 29719505 PMC5913322

[B260] Zhan F. Colla S. Wu X. Chen B. Stewart J. P. Kuehl W. M.. (2007). CKS1B, overexpressed in aggressive disease, regulates multiple myeloma growth and survival through SKP2-and p27Kip1-dependent and-independent mechanisms. Blood 109, 4995–5001. 10.1182/blood-2006-07-038703 17303695 PMC1885527

[B261] Zhang M. Deng Y. Luo Y. Zhang S. Zou H. Cai F.. (2012). Control of BACE1 degradation and APP processing by ubiquitin carboxyl-terminal hydrolase L1. J. Neurochem. 120, 1129–1138. 10.1111/j.1471-4159.2011.07644.x 22212137

[B262] Zhang N.-Y. Tang Z. Liu C.-W. (2008). α-synuclein protofibrils inhibit 26 S proteasome-mediated protein degradation understanding the cytotoxicity of protein pathogenesis. J. Biol. Chem. 283, 20288–20298. 10.1074/jbc.M710560200 18502751

[B263] Zheng C. Geetha T. Babu J. R. (2014). Failure of ubiquitin proteasome system: risk for neurodegenerative diseases. Neurodegener. Dis. 14, 161–175. 10.1159/000367694 25413678

[B264] Zheng Q. Huang T. Zhang L. Zhou Y. Luo H. Xu H.. (2016). Dysregulation of ubiquitin-proteasome system in neurodegenerative diseases. Front. Aging Neurosci. 8:303. 10.3389/fnagi.2016.00303 28018215 PMC5156861

[B265] Zhu C. W. Grossman H. Neugroschl J. Parker S. Burden A. Luo X.. (2018). A randomized, double-blind, placebo-controlled trial of resveratrol with glucose and malate (RGM) to slow the progression of Alzheimer's disease: a pilot study. Alzheimers Dement. 4, 609–616. 10.1016/j.trci.2018.09.009 30480082 PMC6240843

[B266] Zinszner H. Kuroda M. Wang X. Batchvarova N. Lightfoot R. T. Remotti H.. (1998). CHOP is implicated in programmed cell death in response to impaired function of the endoplasmic reticulum. Genes Dev. 12, 982–995. 10.1101/gad.12.7.982 9531536 PMC316680

[B267] Zucchelli S. Marcuzzi F. Codrich M. Agostoni E. Vilotti S. Biagioli M. (2011). Tumor Necrosis factor receptor associated factor 6 (TRAF6) associates with huntingtin protein and promotes its atypical ubiquitination to enhance aggregate formation. J. Biol. Chem. 110:187591 10.1074/jbc.M110.187591 PMC3137084 21454471

